# Deletion of the *Ebf1*, a mouse deafness gene, causes a dramatic increase in hair cells and support cells of the organ of Corti

**DOI:** 10.1242/dev.202816

**Published:** 2024-08-20

**Authors:** Kathryn G. Powers, Brent A. Wilkerson, Kylie E. Beach, Sophie S. Seo, Jose S. Rodriguez, Ashton N. Baxter, Sarah E. Hunter, Olivia Bermingham-McDonogh

**Affiliations:** ^1^ Department of Biological Structure, University of Washington School of Medicine, Seattle, WA 98195, USA; ^2^ Program in Molecular and Cellular Biology, University of Washington, Seattle, WA 98195, USA; ^3^ Department of Otolaryngology-Head and Neck Surgery, Medical University of South Carolina, Charleston, SC 29425, USA

**Keywords:** Cochlear development, Fate specification, Hearing, Inner ear, Supernumery hair cells

## Abstract

Following up on our previous observation that early B cell factor (EBF) sites are enriched in open chromatin of the developing sensory epithelium of the mouse cochlea, we investigated the effect of deletion of *Ebf1* on inner ear development. We used a Cre driver to delete *Ebf1* at the otocyst stage before development of the cochlea. We examined the cochlea at postnatal day (P) 1 and found that the sensory epithelium had doubled in size but the length of the cochlear duct was unaffected. We also found that deletion of *Ebf1* led to ectopic sensory patches in the Kölliker's organ. Innervation of the developing organ of Corti was disrupted with no obvious spiral bundles. The ectopic patches were also innervated. All the extra hair cells (HCs) within the sensory epithelium and Kölliker's organ contained mechanoelectrical transduction channels, as indicated by rapid uptake of FM1-43. The excessive numbers of HCs were still present in the adult *Ebf1* conditional knockout (cKO) animal. The animals had significantly elevated auditory brainstem response thresholds, suggesting that this gene is essential for hearing development.

## Teaser

**Summary:** *Ebf1* deletion in mice causes deafness, aberrant patterning and innervation of sensory cells, and an increase in the number of hair cells in the cochlea.

## INTRODUCTION

The inner ear contains the sensory structures necessary for our sense of both hearing and balance. A single sensory structure mediates hearing, the organ of Corti, which is the sensory region of the cochlear duct. It consists of one row of inner hair cells (iHCs) and three rows of outer HCs (oHCs), all with attendant support cells (SCs) and an extremely regular arrangement along the ventral cochlear duct. The inner border cell (iBC) and inner phalangeal cell (iPhC) are associated with the iHC. iBC projections wrap around the medial face of iHCs, and iPhC projections wrap around the lateral face. Each oHC is associated with a single Deiters' cell (DC). In between these HC/SC groups there are two pillar cells (PCs); the one closest to the iHC is called the inner PC (iPC) and the one closest to the oHCs is called the outer PC (oPC). These PCs form the fluid-filled tunnel of Corti without which the animal cannot hear (Colvin et al., 1996; Hayashi et al., 2007). The development, position and arrangement of these HCs and SCs are dependent on various signaling molecules from multiple families of growth factors expressed in medial (neural) to lateral (abneural) gradients (Driver and Kelley, 2020; Groves and Fekete, 2012). For example, there is a strong expression of BMP4 at the lateral side, which likely restricts the sensory domain in this direction (Ohyama et al., 2010). However, what exactly restricts the development of sensory cells medially is not known. This study investigating the role of EBF1 in developing cochleae suggests that the expression of this transcription factor establishes the medial border of the sensory domain.

In our analysis of the epigenetic regulation of cochlear development, we discovered an enrichment of EBF transcription factor-binding motifs in the open chromatin of SOX2-EGFP prosensory cells (Wilkerson et al., 2019). Other transcription factors known to be important in sensory development were also found. This suggests that a member of the EBF family of transcription factors is likely to play a role in the development of the organ of Corti. We looked at single cell RNA-seq obtained from developing cochleae at the same ages as the ATAC-seq samples, and discovered that EBF1 is likely the EBF family member involved. Indeed, the expression of *Ebf1* in the inner ear was first described by Davis and Reed (1996) and later revealed in the Allen Brain Atlas.

EBF1 is a member of the COE (Collier, OLF1 and EBF1-4) family of transcription factors. The EBFs are helix-loop-helix proteins containing an unusual Zn coordinating domain that binds to DNA. Ebf genes are expressed in many parts of the nervous system and frequently co-expressed with other family members. Mutations in a single Ebf gene can often be compensated for by another family member; thus, loss of function of a single Ebf gene reveals a phenotype if it is the only member of the family expressed. This appears to be the case in the developing cochlear epithelium, as there is little to no expression of *Ebf2*, *Ebf3* and *Ebf4*.

In this study, we examine the effect of deleting *Ebf1* from the otic epithelium on the developing cochlea using a tissue-restricted Cre or a tamoxifen-inducible Cre. We find that there is a dramatic increase of both iHCs and oHCs, and their attendant SCs in the mice with the conditional deletions. The mutant mice also have ectopic patches of sensory epithelia that develop in Kölliker's organ, suggesting that EBF1 is suppressing sensory development in this medial region. The expanded sensory domain is innervated, but the outer spiral bundles do not develop normally in the *Ebf1* mutant mouse. The ectopic HCs we see in the non-sensory Kölliker's organ are also innervated and are surrounded by SCs. Interestingly these extra iHCs and oHCs are retained in the adult cochlea; however, the mice are severely hearing impaired.

## RESULTS

### *Ebf1* is expressed in Kölliker's organ, prosensory cells, HCs and SCs of the developing cochlea

**Fig. 1.**
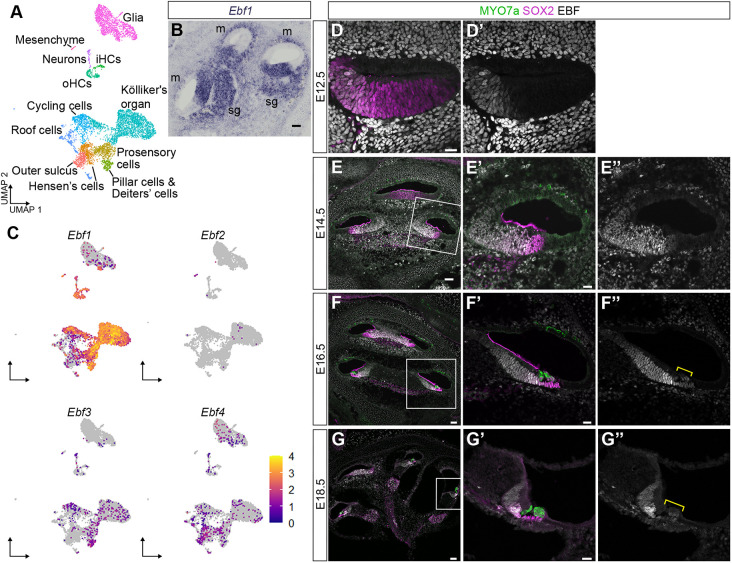
***Ebf1* expression in the developing cochlea.** (A) A uniform manifold approximation and projection (UMAP) plot of SOX2-EGFP FAC-sorted single cells pooled from E12, E14 and E16 cochlear ducts. Cell types were identified based on marker gene expression. (B) *In situ* hybridization shows robust *Ebf1* expression in the ventral cochlear duct in addition to the spiral ganglion (sg) and adjacent mesenchyme (m) by E14.5. (C) Feature plots of pooled E12, E14 and E16 single cells highlighting *Ebf1-4* expression. (D-G″) Confocal images of E12.5, E14.5, E16.5 and E18.5 C57BL/6 cochlear sections immunolabeled with pan-EBF antibody. (E′-G″) Zoomed view of base in cochlear sections (outlined in E-G). (D,D′) At E12.5, EBF protein expression overlaps with the medial domain for SOX2 expression. (E′,E″) In the base of E14.5 cochleae, EBF expression overlaps with SOX2 and extends medially into Kölliker's organ. (F′,F″) In the base of E16.5 cochleae, EBFs are most strongly expressed in the lateral edge of Kölliker's organ, and show reduced expression in HCs, SCs and the developing inner sulcus. (G′,G″) In the base of E18.5 cochlea, EBFs maintain strong expression in the lateral edge of Kölliker's organ medial to the iHC. EBFs appear to be downregulated in the SCs and HCs (brackets in F″ and G″), particularly iHCs. Scale bars: 20 µm for D,D′,E′,E″,F′,F″,G′,G″; 50 µm for E,F,G.

Recent work from our lab identified enrichment of EBF-binding motifs in the open chromatin of cochlear prosensory cells collected at key developmental time points (Wilkerson et al., 2019). To investigate Ebf gene expression in the developing cochlear sensory epithelium, we performed single cell RNA-seq on SOX2-EGFP^+^ cells FAC-sorted from E12, E14 and E16 cochlear ducts. Clustering the cells after sequencing using Seurat allowed us to identify the various cell types present in the sample. We were able to capture the major cell types (prosensory cells, iHCs, oHCs, SCs, neurons, glia, Kölliker's organ cells, cycling cells, roof cells, mesenchymal cells and outer sulcus cells) present during these three developmental time points (Fig. 1A). Our analysis of the SOX2-EGFP^+^ single cells revealed that *Ebf1* is expressed in the developing cochlear epithelium whereas *Ebf2*, *Ebf3* and *Ebf4* show little to no expression (Fig. 1C). More specifically, *Ebf1* is strongly expressed in prosensory cells, iHCs, oHCs, SCs, neurons, Kölliker's organ cells, cycling cells and mesenchymal cells (Fig. 1A,C). *In situ* hybridization of the E14.5 inner ear confirmed our single cell results. *Ebf1* transcript expression was detected in the ventral cochlear duct, spiral ganglion and mesenchyme (Fig. 1B).

Our transcript analyses indicated that *Ebf1* is the primary contributor to EBF protein expression in the developing cochlear sensory epithelium (Fig. 1A-C). To further explore the timing and localization of EBF expression, immunolabeling was performed using a pan-EBF antibody. At E12.5, EBF protein is expressed in a band that overlaps with the medial edge of the expression domain for SOX2 (Fig. 1D,D′), a well-known prosensory marker (Kiernan et al., 2005b). At E14.5, EBF protein expression extends medially into Kölliker's organ and overlaps with the SOX2 domain (Fig. 1E-E″). By E16.5, MYO7a^+^ HCs can be seen in the base and middle of the cochlear sensory epithelium (Sahly et al., 1997). At this stage, EBF protein is most strongly expressed in the population of SOX2^+^ Kölliker's organ cells immediately medial to the iHC row (Fig. 1F-F″) and shows comparatively weak expression in HCs and SCs of the sensory domain (bracket in Fig. 1F″). Although the developing cochlea maintains strong expression of EBF proteins in Kölliker's organ by E18.5 (Fig. 1G-G″), expression is diminished in the sensory cells, particularly in iHCs (bracket in Fig. 1G″). We continue to see EBF immunolabeling in the DCs and iPhCs of the adult organ of Corti (see below).

### Deletion of Ebf1 in the otic vesicle at E9.5 leads to dramatic expansion in the sensory domain of the cochlea and the formation of ectopic sensory patches in Kölliker's organ

To investigate the role of EBF1 in cochlear development, we generated a cKO mouse in which the *Slc26a9* promoter directs Cre-mediated excision of floxed *Ebf1* exons 6-16 (Vilagos et al., 2012). SLC26A9 is a chloride transporter present at E9.5 in the otic vesicle that gives rise to the inner ear epithelium (Urness et al., 2020). Unlike the *Ebf1* global knockout (KO), which is postnatal lethal (Nelson et al., 2019), *Slc26a9^Ebf1-cKO^* mice are fertile and survive into adulthood. The cochleae of mice with the conditional deletion lose EBF immunolabeling in the cochlear epithelium but maintain EBF expression in the mesenchyme (Fig. S1), likely due to *Ebf3* and *Ebf4*, which show low expression levels in the otic epithelium in our single cell analyses (Fig. 1C).

**Fig. 2.**
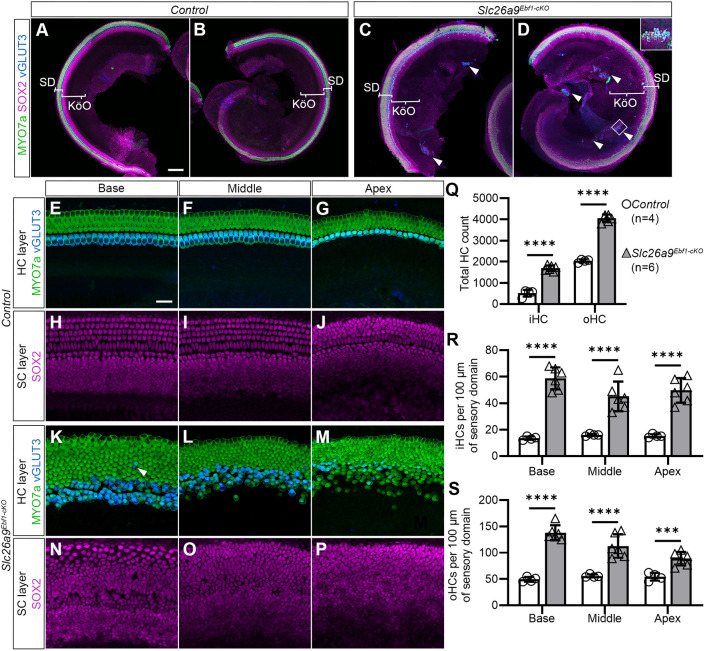
**Deletion of *Ebf1* in the otic vesicle at E9.5 leads to dramatic sensory expansion.** (A,B) Confocal images of basal and apical turns from a P1 littermate control cochlear wholemount. Within the sensory domain (SD), control cochleae exhibit a single row of iHCs (vGLUT3^+^ and MYO7a^+^) medial to three rows of oHCs (vGLUT3^-^ and MYO7a^+^). (C,D) Confocal images of basal and apical turns from a *Slc26a9^Ebf1-cKO^* P1 cochlear wholemount. In addition to three to five discontinuous rows of iHCs medial to approximately six rows of oHCs in the sensory domain, *Slc26a9^Ebf1-cKO^* mice exhibit ectopic sensory patches (arrowheads) throughout Kölliker's organ (KöO). (E-J) Confocal images of the HC and SC layers in the base, middle and apex of a P1 control cochlear wholemount. (K-P) Confocal images of the HC and SC layers in the base, middle and apex of a P1 *Slc26a9^Ebf1-cKO^* cochlear wholemount. A ‘confused’ iHC (arrowhead) can occasionally be seen in the oHC region of *Slc26a9^Ebf1-cKO^* cochlear ducts. (Q) The mean (±s.d.) total iHC and oHC counts in *Slc26a9^Ebf1-cKO^* P1 mice are significantly greater than those for littermate controls (Table S1). (R) Compared with littermate control P1 mice, mean (±s.d.) iHC density in *Slc26a9^Ebf1-cKO^* mice is significantly greater in the base, middle and apex (Table S1). (S) Compared with littermate control P1 mice, mean (±s.d.) oHC density in *Slc26a9^Ebf1-cKO^* mice is significantly greater in the base, middle and apex (Table S1). Sample size: four control and six *Slc26a9^Ebf1-cKO^* mice from two litters, one duct per mouse. Scale bars: 100 µm for A-D; 20 µm for E-P).

To determine the effects of loss of EBF1 in the developing cochlea, we analyzed P1 cochlear wholemounts of control and *Slc26a9^Ebf1-cKO^* mice. We found a dramatic expansion of the sensory epithelium in the mutant mice (Fig. 2). Unlike control littermates with the expected single row of iHCs (vGLUT3^+^ and MYO7a^+^) and three rows of oHCs (vGLUT3^-^ and MYO7a^+^; Fig. 2E-G), mice with the conditional deletion possess three to five rows of iHCs medial to approximately six rows of oHCs (Fig. 2K-M). *Slc26a9^Ebf1-cKO^* cochlear ducts exhibit approximately threefold and twofold increases, respectively, in iHC and oHC counts by P1 (Fig. 2Q). *Slc26a9^Ebf1-cKO^* neonates also demonstrate a delayed base-to-apex sweep of vGLUT3 expression relative to littermate controls (Fig. 2A,B versus C,D), suggesting a developmental delay in cochlear maturation. However, the lengths of P1 *Slc26a9^Ebf1-cKO^* and littermate control cochlear ducts are not significantly different (*P*>0.05, Welch's *t*-test, 4598.1±493.6 µm for nine *Slc26a9^Ebf1-cKO^* mice and 4653.2±349.7 µm for nine littermate controls from four litters; one duct per mouse).

To determine whether the extent of HC expansion varies along the length of the cochlear duct in the mutant mice, we quantified HC density in the base, middle and apex of cochlear wholemounts. Compared with littermate controls, iHC and oHC densities are significantly greater in all regions of the *Slc26a9^Ebf1-cKO^* samples (Fig. 2R,S and Table S1). Although the mutants show an overall increase in HC density across all regions, the increase was greatest at the base for both iHCs and oHCs (Table S1). The increases in HCs are accompanied by defects in either stereocilia bundle or HC orientation. The supernumerary iHCs in the mutant are arranged on either side of the space presumably formed by the developing tunnel of Corti (Fig. 2K). Although we did not quantify the support cell increases, there was a clear expansion of the SOX2^+^ domain (Fig. 2H-J versus N-P)

In addition to an excess of sensory cells within the sensory domain, the *Slc26a9^Ebf1-cKO^* mice also possess ectopic sensory patches throughout their Kölliker's organs (arrowheads in Fig. 2C,D). These ectopic patches resemble miniature vestibular organs, consisting of vGLUT3^+^ HCs resting on a bed of SCs that do not express the markers characteristic of sensory domain SCs (magnified inset capturing the ectopic patch outlined in Fig. 2D and Fig. S2). *Slc26a9^Ebf1-cKO^* supernumerary and ectopic patch HCs have functioning mechanoelectrical transduction (MET) channels, as demonstrated by their uptake of FM1-43 dye (Fig. S3).

### Increases in HC numbers in the *Slc26a9^Ebf1-cKO^* mice are accompanied by increases in SCs

**Fig. 3.**
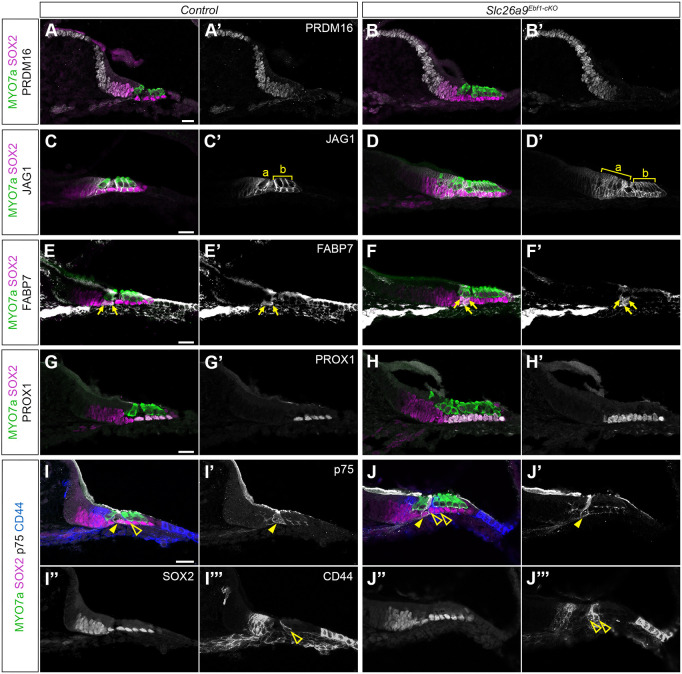
***Slc26a9^Ebf1-cKO^* mice demonstrate increases in SC subtypes.** (A-J‴) Sections capturing the base of P1 control and *Slc26a9^Ebf1-cKO^* cochleae. (A-B′) Significantly more PRDM16^+^ nuclei reside in Kölliker's organ in *Slc26a9^Ebf1-cKO^* cochleae compared with littermate controls. (C-D′) JAG1^+^ SCs are increased in *Slc26a9^Ebf1-cKO^* cochlear sections and continue to extend projections around iHCs (bracket a) and oHCs (bracket b). (E-F′) A pair of FABP7^+^ iBCs/iPhCs (arrows) are seen in control cochlear sections. More than two FABP7^+^ iBCs/iPhCs are present in *Slc26a9^Ebf1-cKO^* cochlear sections. (G-H′) *Slc26a9^Ebf1-cKO^* cochleae demonstrate increases in PROX1^+^ PC and DC nuclei. (I-J‴) A single p75^+^ iPC (closed yellow arrowheads) can be observed in both control and *Slc26a9^Ebf1-cKO^* cochlear sections. Multiple CD44^+^ oPCs (open arrowheads) are present in *Slc26a9^Ebf1-cKO^* cochlear sections, whereas only a single oPC is observed in littermate controls. The expression domain of SOX2 protein shows dramatic expansion in the *Slc26a9^Ebf1-cKO^* cochleae compared with littermate controls. Scale bars: 20 µm (bars in A,C,E,G,I apply to A-B′,C-D′,E-F′,G-H′ and I-J‴, respectively).

Supernumerary HCs are neighbored by supernumerary SCs (Fig. 2N-P), and mutant mice exhibit more nuclear layers in Kölliker's organ than littermate controls (Fig. 3A,A′ versus B,B′). We measured the areas of the developing sensory domain and Kölliker's organ in the base of mid-modiolar sections collected from P1 *Slc26a9^Ebf1-cKO^* and littermate control mice. Anti-SOX2 antibody was used to visualize the developing sensory domain in the base of mid-modiolar sections collected from P1 *Slc26a9^Ebf1-cKO^* and littermate control mice (Fig. 3). As expected, we found that the area of SOX2 expression was significantly greater in *Slc26a9^Ebf1-cKO^* than littermate control sections (*P*<0.05, 2534±682 µm^2^ for *Slc26a9^Ebf1-^*^cKO^ and 1751±331 µm^2^ for littermate controls; Table S1). Similarly, we used a marker unique to Kölliker's organ, PRDM16, to assess changes in the overall size of this transient structure (Ebeid et al., 2022). We found that the area of PRDM16^+^ expression was significantly greater in *Slc26a9^Ebf1-cKO^* than in littermate control sections (*P*<0.05, 4391±940 µm^2^ for *Slc26a9^Ebf1-^*^cKO^ and 3146±398 µm^2^ for littermate controls; Table S1). Kölliker's organ appears to increase in size, along with the sensory domain, in the mutant mice.

To better characterize the increase in SCs of the sensory domain, we used several markers to identify SC subtype-specific changes in patterning within the base of P1 mid-modiolar sections. In addition to anti-SOX2 antibody, anti-jagged1 (JAG1) antibody was used to examine overall SC expansion. At P1, JAG1 is expressed in sensory domain SCs and lateral Kölliker's organ cells, and SOX2 expression overlaps with JAG1 and extends more medially into Kölliker's organ. The expression domain of JAG1 is expanded in cochlear sections from the mutant mice compared with littermate controls (Fig. 3C,C′ versus D,D′). To determine which SC subtypes contribute to the observed SC expansion in the mutant mice, we used specific markers for iBCs, iPhCs, iPCs, oPCs and DCs. When compared with littermate controls, the mutant mice exhibited an increase in anti-FABP7 labeled iBCs/iPhCs (arrows in Fig. 3E,E′ versus F,F′) and anti-PROX1 labeled PCs and DCs (Fig. 3G,G′ versus H,H′). Further analysis with anti-p75 and anti-CD44 antibodies, which label iPCs and oPCs (Hertzano et al., 2010; Knipper et al., 1996), respectively, indicates that increases in oPC and DC numbers likely account for the observed increases in PROX1^+^ nuclei. Although multiple CD44^+^ oPCs can be seen in *Slc26a9^Ebf1-cKO^* cochlear sections, only one p75^+^ (NGFR) iPC is typically seen (arrowheads in Fig. 3J-J‴). Cells lateral to the oHCs do not demonstrate changes in their numbers. FABP7 is expressed by Hensen's cells (HeCs) in addition to iBCs and iPhCs, and CD44 is expressed by Claudius cells (CCs) in addition to oPCs. Compared with littermate control cochlear sections, HeC and CC populations are unchanged in *Slc26a9^Ebf1-cKO^* cochlear samples (Fig. 3E,E′,I,I‴ versus F,F′,J,J‴).

**Fig. 4.**
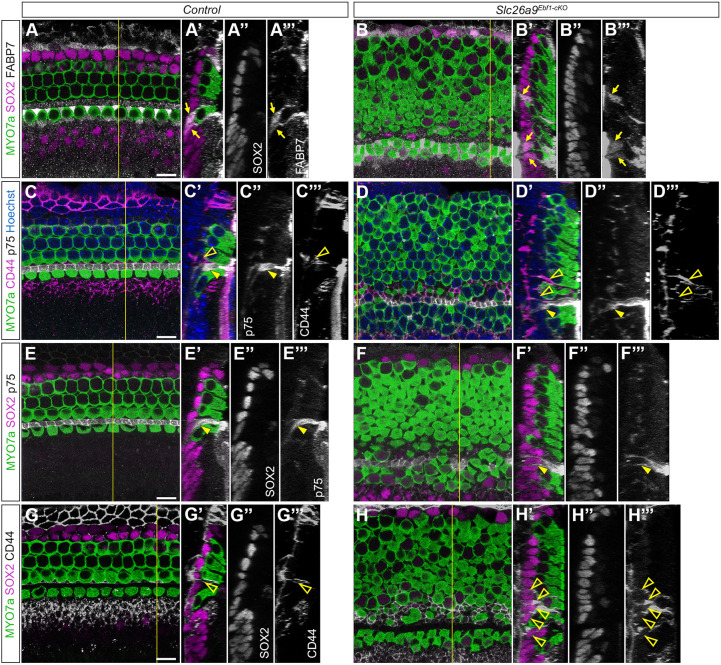
**Supernumerary iHCs continue to direct SC patterning.** (A-H) Single *xy* plane confocal images of basal region of P1 control and *Slc26a9^Ebf1-cKO^* cochlear wholemounts. (A′-H′) Orthogonal projections of the *yz* plane (yellow lines in *xy* plane images) for the control and *Slc26a9^Ebf1-cKO^* cochlear wholemounts. (A-B‴) FABP7^+^ iBCs/iPhCs (arrows) are arranged in two rows in control cochleae. iBCs form a single row on the medial side of the iHCs and iPhCs form a single row on the lateral side. Supernumerary iHCs in *Slc26a9^Ebf1-cKO^* cochleae appear to be adjacent to at least one FABP7^+^ iBC/iPhC. These supernumerary FABP7^+^ iBCs/iPhCs are present in two to six discontinuous rows. (C-F‴) p75^+^ iPCs (closed yellow arrowheads) are arranged in a single continuous row in control cochleae. Although p75^+^ iPCs are still arranged in a single row in *Slc26a9^Ebf1-cKO^* cochleae, this row is interrupted by supernumerary HCs. (C-D‴,G-H‴) CD44^+^ oPCs (open arrowheads) are arranged in a single continuous row in control cochleae (C-C‴,G-G‴). These cells, by contrast, are arranged in at least two discontinuous rows in *Slc26a9^Ebf1-cKO^* cochleae (D-D‴,H-H‴). Scale bars: 10 µm (bars in A,C,E,G apply to A-B‴,C-D‴,E-F‴ and G-H‴, respectively).

To further investigate the effects of *Ebf1* deletion in patterning of medial SCs, we performed immunolabeling on P1 cochlear wholemounts. Anti-FABP7 labeling revealed that mice with a conditional deletion of *Ebf1* possess more than the typical two rows of iBCs/iPhCs (arrows in Fig. 4A-A‴ versus B-B‴). In the mutant cochlea, FABP7^+^ iBCs/iPhCs reside next to the medial and/or lateral faces of supernumerary HCs that resemble iHCs in their cell shape and position along the medial-lateral axis (arrows in Fig. 4B-B‴). The occasional iHC can be observed in the oHC region (Figs 2K and 4B-B‴). Even among the oHCs, this iHC continues to direct patterning of a FABP7^+^ iBCs/iPhCs (Fig. 4B-B‴). Like iBCs/iPhCs, *Slc26a9^Ebf1-cKO^* cochlear ducts possess more CD44^+^ oPCs than littermate controls. Although controls possess a single continuous row of oPCs lateral to the row of iPCs (Fig. 4C-C″,G-G‴), *Slc26a9^Ebf1-cKO^* samples exhibit at least two discontinuous rows of oPCs lateral to their iPCs (Fig. 4D-D‴,H-H‴). Although the *Ebf1* deletion induces an increase in many types of SCs, it does not appear to increase iPC numbers. p75^+^ iPCs are present in a single row in both littermate control and *Slc26a9^Ebf1-cKO^* cochlear ducts (Fig. 4C-C‴,E-E‴ versus D-D‴,F-F‴).

### Loss of Ebf1 expression leads to prolonged prosensory cell proliferation and delayed sensory cell differentiation

**Fig. 5.**
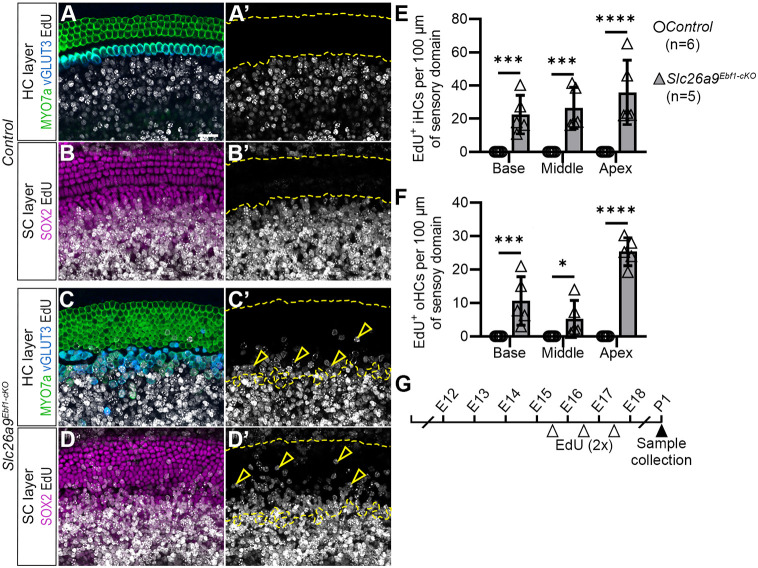
**Prolonged prosensory cell proliferation in *Slc26a9^Ebf1-cKO^* cochleae*.*** (A-D′) Confocal images of the HC and SC layers in the middle of EdU-labeled P1 control and *Slc26a9^Ebf1-cKO^* cochlear wholemounts. (A-B′) Control HCs and underlying SCs (dashed yellow lines) lack EdU labeling. (C-D′) *Slc26a9^Ebf1-cKO^* HCs and underlying SCs are EdU^+^ (open arrowheads in C′ and D′). (E) Compared with littermate control P1 mice, mean (±s.d.) EdU^+^ iHC density in *Slc26a9^Ebf1-cKO^* mice is significantly greater in the base, middle and apex (Table S1). (F) Compared with littermate control P1 mice, mean (±s.d.) EdU^+^ oHC density in *Slc26a9^Ebf1-cKO^* mice is significantly greater in the base and apex (Table S1). (G) Mice used in proliferation experiments were treated with EdU twice a day for 3 days (E15.5-E17.5) and collected at P1. Sample size: six control and five *Slc26a9^Ebf1-cKO^* mice from two litters, one duct per mouse. Scale bars: 20 µm (bar in A applies to all panels).

The increase in sensory cell numbers after *Ebf1* deletion suggests that the proliferation of the sensory progenitors may be prolonged in the mutant mice. To assess whether loss of EBF1 leads to prosensory cell proliferation beyond the typical E12-E15 window (Ruben, 1967), we performed EdU labeling on pregnant dams carrying *Slc26a9^Ebf1-cKO^* and control littermates. EdU was administered twice a day for 3 days starting on E15.5, and cochlear wholemounts were analyzed at P1 (Fig. 5G). Although HCs and attendant SCs fail to incorporate EdU in control cochleae (Fig. 5A-B′), many HCs and SCs in the sensory domains of *Slc26a9^Ebf1-cKO^* are EdU^+^ (open arrowheads in Fig. 5C-D′). Quantitative analysis of EdU^+^ iHC and oHC density revealed that the cochleae of the mutant mice possess significantly more EdU^+^ HCs than samples from littermate controls (Fig. 5E,F and Table S1). The increase in EdU^+^ HCs is present in the *Slc26a9^Ebf1-cKO^* samples throughout the extent of the cochlea, with more EdU^+^ oHCs in the apex followed by the base and middle of *Slc26a9^Ebf1-cKO^* cochlear ducts (Table S1). These results suggest that prolonged cell proliferation in the prosensory cells likely contributes to the sensory expansion seen after *Ebf1* deletion.

The precise coordination of cell cycle exit and sensory cell differentiation are essential for cochlear development (Benito-Gonzalez and Doetzlhofer, 2014; Driver and Kelley, 2020). To assess whether delayed cell cycle exit in *Slc26a9^Ebf1-cKO^* mice is accompanied by delayed differentiation, we measured the sweep of MYO7a expression at E16.5 (Fig. S4), well after the onset of HC differentiation (Chen et al., 2002). The distance from the apex to the first MYO7a^+^ cell is significantly greater in *Slc26a9^Ebf1-cKO^* cochlear ducts than in control cochlear ducts. This difference is not due to impaired formation of the SOX2^+^ prosensory domain, which suggests that the sweep of differentiation is delayed in *Slc26a9^Ebf1-cKO^* samples (Fig. S4A″,B″,C). At E16.5, it is worth mentioning that the lengths of *Slc26a9^Ebf1-cKO^* and control cochlear ducts are significantly different, suggesting that convergent extension is delayed (*P*<0.01, 3724±120 µm for *Slc26a9^Ebf1-cKO^* and 4463±287 µm for littermate controls; Table S1). By P1, *Slc26a9^Ebf1-cKO^* and littermate control ducts are similar in length.

### Supernumerary HCs and SCs survive in adult *Slc26a9^Ebf1-cKO^* mice

**Fig. 6.**
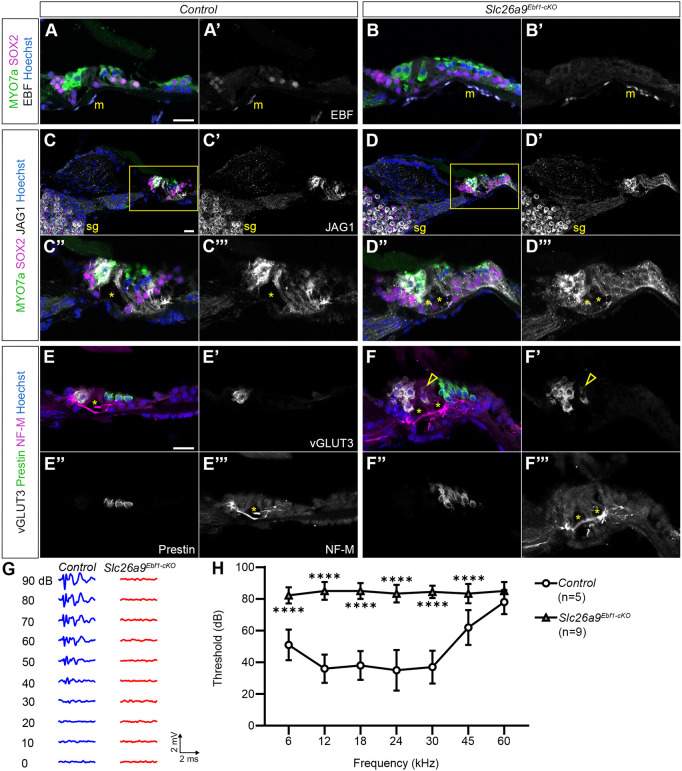
**Supernumerary HCs and SCs are present in adult *Slc26a9^Ebf1-cKO^* mice, which are deaf.** (A-F‴) Sections capturing the middle of P60 control and *Slc26a9^Ebf1-cKO^* cochleae. (C″-D‴) Zoomed views of the organ of Corti (outlined in C and D). (A-B′) Ebf expression is maintained in the SC and mesenchymal cell nuclei of adult control cochleae. Ebf expression is not evident in the sensory epithelium of *Slc26a9^Ebf1-cKO^* cochleae but can still be seen in the mesenchyme (m). (C-F‴) Although the expected single tunnel of Corti is observed in controls, multiple tunnels of Corti (asterisks) are present in *Slc26a9^Ebf1-cKO^* cochlear sections. (C-D′) JAG1 expression is present in the spiral ganglion (sg) and in SCs of control and *Slc26a9^Ebf1-cKO^* cochleae. (C″-D‴) JAG1^+^ supernumerary SCs persist in *Slc26a9^Ebf1-cKO^* cochlear sections. (E-F‴) *Slc26a9^Ebf1-cKO^* supernumerary iHCs are vGLUT3^+^ and supernumerary oHCs are prestin^+^. The occasional vGLUT3^+^ iHC can be seen in the PC region (open arrowheads). NF-M^+^ neuronal projections extend to supernumerary iHCs and oHCs in adult samples. (G) Example control and *Slc26a9^Ebf1-cKO^* ABR waveforms generated in response to broadband click stimulus. (H) P50-P74 *Slc26a9^Ebf1-cKO^* mice demonstrate elevated ABR thresholds compared with littermate controls (Table S1). Sample size: five controls (one female and four males) and nine *Slc26a9^Ebf1-cKO^* mice (six females and three males). Scale bars: 20 μm in A-C (bars apply to A-A‴, B-B‴ and C-C‴, respectively); 9 μm in C″,C‴ and D″,D‴.

Our results on the developing cochlea show dramatic sensory expansion in mice with a conditional deletion in *Ebf1*; to assess whether this phenotype persists in adult mice, we performed immunolabeling on mid-modiolar cochlear sections collected from P60 *Slc26a9^Ebf1-cKO^* and littermate control mice. Much like *Slc26a9^Ebf1-cKO^* neonatal cochleae (Fig. S1B,B′), adult *Slc26a9^Ebf1-cKO^* cochleae continue to express EBFs in their mesenchymal cells and show little to no EBF expression in the cochlear sensory epithelium (Fig. 6B,B′). Control littermates exhibit EBF expression in their SCs in addition to their mesenchymal cells (Fig. 6A,A′). To examine the effects of *Ebf1* deletion in SC survival and maturation in the adult mice, we looked at expression of several HC and SC markers. We found that in adult mutant mice, the increase in SC numbers persisted and, strikingly, the additional SCs appear to give rise to multiple tunnels of Corti (asterisks in Fig. 6D″,D‴,F,F‴). To assess HC survival and maturation, we performed labeling using anti-vGLUT3 and anti-prestin antibodies. We found that there was a loss in oHCs in the base of a subset of adult *Slc26a9^Ebf1-cKO^* cochleae; however, supernumerary HCs appear to survive throughout the rest of the duct. Adult supernumerary vGLUT3^+^ iHCs are typically found medial to tunnels of Corti, although the occasional vGLUT3^+^ iHC can be seen in the PC region (open arrowhead in Fig. 6F,F′). Neurofilament (NF-M) labeling indicates that supernumerary iHCs and oHCs are innervated (Fig. 6F‴). Interestingly, *Slc26a9^Ebf1-cKO^* mice demonstrate significantly elevated auditory brainstem response (ABR) thresholds for all tested stimulus frequencies, except 60 kHz. These results suggest that adult *Slc26a9^Ebf1-cKO^* mice are deaf (Fig. 6G,H).

### Loss of Ebf1 expression leads to aberrant innervation in the sensory domain and ectopic sensory patches are innervated

**Fig. 7.**
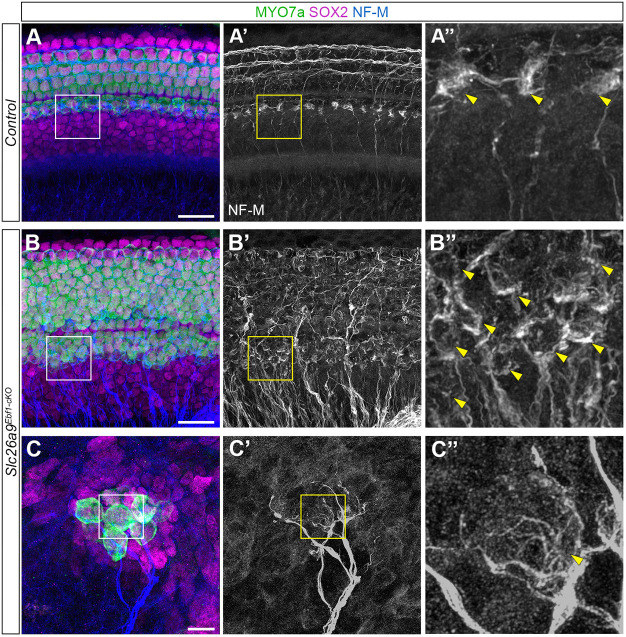
**Aberrant innervation of *Slc26a9^Ebf1-cKO^* HCs.** (A-B′) The basal region of P1 control and *Slc26a9^Ebf1-cKO^* cochlear wholemounts. (C,C′) An ectopic sensory patch near the apex of a *Slc26a9^Ebf1-cKO^* cochlear wholemount. (A″,B″) Zoomed views of NF-M^+^ neuronal projections extending to and from the control and *Slc26a9^Ebf1-cKO^* iHC regions (outlined in A-B′). (C″) Zoomed view of NF-M^+^ neuronal projections extending to and from ectopic HCs (outlined in C and C′). (A-A″) Numerous fibers terminate on control iHCs (arrowheads in A″). Neuronal projections innervate multiple oHCs within the same row, forming three spiral bundles in control cochleae. (B-B″) Fibers continue to terminate on supernumerary HCs (arrowheads in B″). Organization of spiral bundles is lost in *Slc26a9^Ebf1-cKO^* cochleae. (C-C″) HCs in ectopic sensory patches are innervated (arrowhead in C″). Scale bars: 20 µm for A-B′; 10 µm C,C′; 4.6 μm in A″,B″; 2.3 μm in C″ (bars in A-C apply to A-A″, B-B″ and C-C″, respectively).

HCs are known to secrete neurotrophins that direct their innervation by spiral ganglion neurons (Fariñas et al., 2001; Fritzsch et al., 1997). To determine whether supernumerary HCs are innervated, we performed immunolabeling with anti-NF-M antibodies. P1 littermate control mice demonstrate the typical innervation pattern. Numerous neuronal projections terminate on iHCs (arrowheads in Fig. 7A″), and the type II afferent projections turn towards the base, innervating multiple oHCs in the same row and forming three spiral bundles (Fig. 7A,A′) (Liberman et al., 1990). Although fibers continue to terminate on *Slc26a9^Ebf1-cKO^* iHCs (arrowheads in Fig. 7B″), the spiral bundles are completely lost in the oHC region and the innervation appears disorganized (Fig. 7B,B′). In addition to fibers extending to supernumerary HCs in sensory domain, HCs in the ectopic sensory patches of Kölliker's organ are also innervated (Fig. 7C-C″).

### Deletion of Ebf1 in the developing sensory epithelium at E11 leads to moderate expansion in the sensory domain

**Fig. 8.**
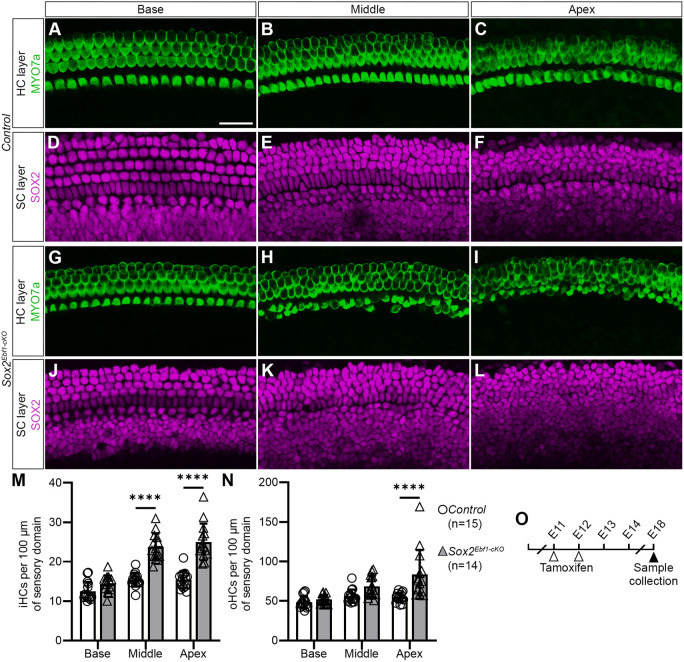
**Deletion of *Ebf1* in the prosensory domain at E11 and E12 leads to moderate sensory expansion.** (A-F) Confocal images of the HC and SC layers the base, middle and apex of an E18 *Sox2^CreER^*-negative control cochlear wholemount after tamoxifen injection at E11 and E12. (G-L) Confocal images of the HC and SC layers in the base, middle and apex of an E18 *Sox2^Ebf1-cKO^* cochlear wholemount. (M) Compared with E18 *Sox2^CreER^*-negative littermate controls, mean (±s.d.) iHC density in *Sox2^Ebf1-cKO^* mice is significantly greater in the middle and apex (Table S1). (N) Compared with E18 *Sox2^CreER^*-negative littermate controls, mean (±s.d.) oHC density in *Sox2^Ebf1-cKO^* mice is significantly greater in the apex (Table S1). (O) *Sox2^CreER^ Ebf1^fl/fl^* and *Ebf1^fl/fl^* littermates were treated with tamoxifen at E11 and E12 before being collected for analysis at E18. Sample size: 15 *Sox2^CreER^*-negative controls and 14 *Sox2^Ebf1-cKO^* mice from six litters, one duct per mouse. Scale bar: 50 µm (bar in A applies to all panels).

The *Slc26a9^Ebf1-cKO^* mice show the effects of the loss of the *Ebf1* gene at a very early stage of cochlear development (E9.5). To determine the role of EBF1 at later stages of development, we used an inducible Cre-mouse line: *Sox2^CreER^*. This second Cre-line was crossed to *Ebf1^fl/fl^* mice, and then *Ebf1* deletion was induced with two tamoxifen treatments: one at E11 and another at E12. Owing to tamoxifen toxicity (Ved et al., 2019), we opted to analyze these litters at E18 (Fig. 8O). iHCs were distinguished from oHCs based on their position relative to the developing tunnel of Corti. At E18, *Sox2^Ebf1-cKO^* mice show extra iHCs and oHCs in an apically-biased manner. In the apex, iHC density and oHC density are both significantly greater in *Sox2^Ebf1-cKO^* than *Sox2^CreER^*-negative control samples. In the middle, iHC density is greater in *Sox2^Ebf1-cKO^* than that in *Sox2^CreER^*-negative controls. *Sox2^Ebf1-cKO^* cochlear ducts show no difference in oHC density in the middle and no differences in HC density in the base (Fig. 8M,N and Table S1). In *Sox2^Ebf1-cKO^* cochlear ducts, most of the extra iHCs are in a second row medial to the main row, and instances of oHCs in a fourth row could be observed. Alignment of oHC rows is imperfect in *Sox2^Ebf1-cKO^* samples (Fig. 8G-I). Loss of EBF1 in the developing sensory epithelium at E11/12 does not significantly affect cochlear duct length (*P*>0.05, Welch's *t*-test, 3592.3±581.0 µm for seven *Sox2^Ebf1-cKO^* and 4042.6±476.9 µm for 13 *Sox2^CreER^*-negative controls, five litters; one duct per mouse). To discriminate between the effects of *Ebf1* deletion and *Sox2* haploinsufficiency on HC formation (Atkinson et al., 2018), we compared HC density in *Sox2^CreER^ Ebf1^fl/fl^* mice not treated with tamoxifen with that of *Sox2^CreER^*-negative littermate controls treated with tamoxifen (13.0±0.8 iHCs and 51.8±2.8 oHCs per 100 µm in the base, 16.2±0.4 iHCs and 53.0±2.8 oHCs per 100 µm in the middle, 15.7±0.5 iHCs and 49.3±2.3 oHCs per 100 µm in the apex for three *Sox2^CreER^ Ebf1^fl/fl^* mice not treated with tamoxifen; one duct per mouse). No significant differences were observed in HC density of *Sox2^CreER^ Ebf1^fl/fl^* mice not treated with tamoxifen and the *Sox2^CreER^*-negative control samples (*P*>0.05, two-way ANOVA with Tukey's multiple comparison's test).

## DISCUSSION

### The role of Ebf1 in neural development

EBF1 is best known for its role in B cell lineage development, but it also plays a role in nervous tissues. EBF1 has been shown to be necessary for striatal medium spiny neuron development and differentiation (Lobo et al., 2008). It has also been shown to play a role in dopaminergic neuron development with loss of these cells in the substantia nigra of *Ebf1^-/-^* mice (Yin et al., 2009). In chick neural tube, early neurogenic genes such as *Ngn2* and *NeuroM* have been shown to activate *Ebf1*, allowing neuronal differentiation and migration to take place (Garcia-Dominguez et al., 2003). The olfactory system is the most closely related to the auditory system, and, in this system, *Ebf1* has roles in the regulation of genes in the odorant signaling cascade (Davis and Reed, 1996). Davis and Reed have described *Ebf1* expression in many areas of the nervous system, including the inner ear. In this study, we have systematically described the expression of *Ebf1* in the developing cochlea using immunohistochemistry, *in situ* hybridization and single cell RNA-seq. Although many studies suggest that *Ebf1* plays a role after exit from the cell cycle, we observed expression in areas of the cochlear epithelium that are still in the cell cycle. However, our data are suggestive of a regulation of exit from the cell cycle, because we see a dramatic increase in cells that develop into sensory cells when *Ebf1* is deleted at the otocyst stage.

### Expansion of the sensory domain in the developing cochlea of *Ebf1* cKO mice

The most obvious phenotype in the *Slc26a9^Ebf1-cKO^* is the more than doubling of the sensory epithelium. This result suggests that *Ebf1* plays a role in either regulating proliferation of prosensory cells or in limiting the extent of the developing sensory epithelial domain. Sensory epithelium patterning in the cochlea can be subdivided into two key processes: prosensory domain establishment and sensory cell differentiation (Brown and Groves, 2020). The prosensory domain is defined between E12 and E15 as prosensory cells undergo cell cycle exit in a wave that starts at the apex of the cochlea and sweeps to the base (Ruben, 1967). HC and SC differentiation proceeds in the opposite direction, starting at the base around E14 and reaching the apex during neonatal stages (Chen et al., 2002).

The development of the sensory epithelium is highly regulated and dependent on many signaling pathways (Groves and Fekete, 2012). Changes in several pathways give rise to cochleae with extra HCs. BMP4 is produced by the cells on the lateral edge of the developing organ of Corti, the development of which is dependent on low levels of BMP4 but inhibited by high levels. BMP4 is responsible, at least in part, for the sharp border at the lateral edge of the sensory epithelium (Ohyama et al., 2010). There is a gradient of WNTs and FGF10 from Kölliker's organ on the medial side of the sensory domain. The sensory domain itself is defined by expression of *Fgf20*, which is initially expressed in base and sweeps towards the apex ahead of MYO7a expression (Hayashi et al., 2008). The Notch pathway is also involved in sensory epithelial patterning. During early development, it is responsible for inducing a sensory fate via signaling from JAG1 (Kiernan et al., 2006) expressed in Kölliker's organ and overlapping the region where iHCs will form (lateral induction). Later in development, the Notch pathway suppresses HC fate because of the early expression of Notch ligands by HCs, thus allowing the cells next to each HC to develop as SCs (lateral inhibition). There are numerous mutations in Notch, its ligands or effectors that will give extra HCs (Basch et al., 2016a,b; Kiernan et al., 2005a).

How does EBF1 fit with all these signaling mechanisms? It is clear from our data that there is a delay in cell cycle exit. Other labs have shown that prolonged prosensory cell proliferation leads to increases in both HCs and SCs (Chen and Segil, 1999; Jacques et al., 2012; Kiernan et al., 2005a; Tateya et al., 2011). A doubling in the width of the sensory epithelium could be accounted for by progenitors going through one more cell cycle. This, however, does not account for the more than doubling of the iHCs. We propose that EBF1 plays an early role in regulating exit from the cell cycle and a later role in maintaining the medial border of the sensory epithelium such that the cells closest to the sensory epithelium do not develop as HCs or SCs. The suppression of sensory epithelium formation also accounts for the ectopic sensory patches we see in the cochlea from *Slc26a9^Ebf1-cKO^* animals. This two-stage requirement for EBF1 activity is consistent with our finding that when *Ebf1* is eliminated at E11/12, we only find extra iHCs, whereas oHC numbers are unaffected.

In addition to extra HCs in the *Ebf1* conditional deletions, we also observed extra SCs. The extra HCs in mice with either conditional deletion (*Slc26a9^Ebf1-cKO^* or *Sox2^Ebf1-cKO^*) appear to pattern their immediately adjacent SCs, possibly via a secreted factor. Such a factor may be *Fgf8*, which is uniquely expressed only by iHCs. Mutations that affect the FGF signaling pathway have been shown to lead to both defects in PC differentiation as well as extra oHCs (Hayashi et al., 2007; Shim et al., 2005). The extra iHCs in the *Ebf1* cKOs should lead to a higher concentration of FGF8; previous studies have shown that overexpression of *Fgf8* in explant cultures will induce extra p75^+^ iPCs (Jacques et al., 2007). One might have anticipated that the mutant would have extra iPCs rather than the extra oPCs that we observe in the *Slc26a9^Ebf1-cKO^* mice. Mice with a *Fgfr3* mutation, which causes an overactive kinase activity of the receptor, also have extra oPCs (Mansour et al., 2013). In the *Fgfr3* mutants, it is thought that the receptor is activated by FGF10, which is abundantly expressed in Kölliker's organ.

### Ectopic patches of sensory epithelium present in the Kölliker's organ of *Slc26a9^Ebf1-cKO^*

The number of ectopic sensory patches in Kölliker's organ is highly variable; however, every mutant cochlea examined had at least one. These are located throughout the cochlea and at various positions along the medial axis of Kölliker's organ. We have never seen complete conversion of Kölliker's organ to sensory epithelium, which suggests that there is more than one inhibitor of sensory development present. We also do not know what kind of HCs are in these ectopic patches. The surrounding cells express SOX2, suggesting that they are SCs, but they do not express any of the specific markers for medial SCs (iBCs, iPhCs and PCs). The HCs do express *Slc17a8*, suggesting that these are either iHCs or vestibular HCs. They also show rapid uptake of FM1-43, indicating active MET channels. We have only examined adult cochleae from the *Ebf1* mutants as sections, so we cannot say at this stage whether these ectopic HCs are retained. It appears that the inner sulcus does form at least in the base.

### Deafness

In the *Slc26a9^Ebf1-cKO^* animal, there are many defects in the sensory epithelium. We see extra iHCs, oHCs, iPhCs, oPCs and DCs, as well as defective neural connections, any of which would dramatically change the response to sound of the animal. In the adults, we found that the extra HCs were retained, and the animal showed dramatic hearing loss. There are multiple potential reasons for this result, including the sheer weight of the extra cells causing mechanical disturbances to the propagation of sound waves along the basilar membrane. We also noticed defects in the tectorial membrane, which appeared thinner and more disorganized in the mutant animals, and defects in innervation.

In summary, we have identified EBF1 as a key factor for patterning the mammalian cochlea and deletion of *Ebf1* leads to deafness in mice. We also found expression of *Ebf1* in the vestibular system but see no vestibular phenotype in the cKO mice. Remaining questions relate to the mechanism of action of EBF1 in cochlear development. From what is known about this family, it could be acting as a transcriptional activator, repressor or chromatin remodeler (Ramírez et al., 2010). It may also have different mechanisms that are context dependent at the various stages of development. We are currently looking into the pathways that are mis-regulated when *Ebf1* is deleted in the developing inner ear. In a recent study, the expression of *Ebf1* was shown to be regulated by SOXC family. In a KO of *Sox4* and *Sox11* in the inner ear, the expression of *Ebf1* was dramatically downregulated along with other prosensory factors, such as *Fgf20* (Hayashi et al., 2008; Wang et al., 2023). In addition, EBF1 appears to be regulated by FGF; *Ebf1* is downregulated in the otic mesenchyme but appears to be upregulated in the epithelium at E12.5 in *Fgf9/20* double KO mice (Ebeid and Huh, 2020). The importance of *Ebf1* in cochlear development is underscored by a recent publication that also analyzed a KO and a cKO for *Ebf1* (Kagoshima et al., 2024). The results from this study are similar to ours, although different mutant mice are used. Their findings agree with those we detail in this report and further support an important role for EBF1 in development of the organ of Corti and suggest its mutation might cause deafness in humans.

## MATERIALS AND METHODS

### Mice

Mice were housed in either the University of Washington Department of Comparative Medicine or in the Medical University of South Carolina Division of Lab Animal Resources. All experiments were approved by the Institutional Animal Care and Use Committee of the University of Washington, as well as by the Medical University of South Carolina, and were performed in accordance with the standards outlined by the National Institutes of Health (NIH). Mice were euthanized in accordance with IACUC approved procedures and in line with NIH policies.

*Ebf1^fl/fl^* mice were obtained from Jackson labs on a C57BL/6 background (strain 028104). *Sox2^CreER^* mice obtained from Jackson labs on a mixed background of C57BL/6 and 129S4/SVJae (strain 017593), and the *Slc26a9^P2A-Cre^* mice were obtained on a C57BL/6 background (Urness et al., 2020). To generate *Slc26a9*- and *Sox2*-conditional *Ebf1* knockout mice, *Slc26a9^P2A-Cre^* and *Sox2^CreER^* mice were crossed with *Ebf1^fl/fl^* females to generate *Cre^+^ Ebf1^fl/+^* progeny. Male *Cre^+^ Ebf1^fl/+^* mice identified by genotyping (see below) were then crossed with *Ebf1^fl/fl^* mice to generate litters containing *Cre^+^ Ebf1^fl/f^*^l^ mice. *Cre^+^ Ebf1^fl/fl^* mice identified by genotyping were bred with *Ebf1^fl/fl^* mice to generate *Cre^+^ Ebf1^fl/f^*^l^ and *Ebf1^fl/fl^* littermates for our phenotypic analyses. To induce Cre recombination, timed-pregnant dams carrying *Sox2^CreER^ Ebf1^fl/fl^* and *Ebf1^fl/fl^* embryos received tamoxifen diluted in corn oil (100 mg/kg; Sigma-Aldrich T5648) via oral gavage at E11 and at E12. *Sox2-EGFP* knock-in mice (Arnold et al., 2011) from Jackson labs (strain 017592) were bred to generate time-pregnant litters. *Sox2-EGFP^+^* embryos were identified by epifluorescence and their cells were purified by FAC-sorting for single cell analysis. Stages were verified by Theiler's criteria. Tail tips or ear punches were collected for genotyping.

Mice positive for *Sox-CreER* were genotyped with the following primer pair: TCCTTAGCGCCGTAAATCAA and TGCCAGGATCAGGGTTAAAG. Mice positive for *Slc26a9^P2A-Cre^* were genotyped using the following primer trio: GGAGGAACACAGTTCACAGT, GTGTCTGGTGTGGCTGATGACC and ATGGGTTCACCAGAGTCTCATC. *Ebf1^fl/fl^* mice were genotyped using two distinct primer pairs: (1) TGTGGCAACCGAAATGAG and CCTGTGAGCGACACAAAGC, in addition to (2) ACGACTTCTTCAAGTCCGCC and TCTTGTAGTTGCCGTCGTCC. The first primer pair was designed to identify the presence of the wild-type *Ebf1* allele (Vilagos et al., 2012). The second primer pair was used to reveal the presence of the *EGFP* associated with the fusion protein encoded in the floxed *Ebf1* allele (Vilagos et al., 2012). Mice were identified as *Ebf1^fl/fl^* if they tested negative for wild-type *Ebf1* allele and positive for *EGFP*.

### *In situ* hybridization

The hybridization was performed as described previously (Hayashi et al., 2007; Wilkerson et al., 2021). Briefly E14.5 heads were fixed in a modified Carnoy's solution overnight at 4°C. Heads were then dehydrated using increasing concentrations of ethanol and left in 100% ethanol overnight at 4°C. Heads were washed in xylene twice before embedding in low melt paraffin (Thermo Fisher Scientific, 23-021-401) and sectioning at 8 mm. Mouse *Ebf1* cDNA was obtained from the Allen Institute (Seattle, WA, USA). Digoxigenin (DIG)-labeled probes for *Ebf1* were generated using SP6 RNA polymerase (Promega P1085) and the following nested PCR primers: CTCAGTCACCACAAGCATGAAT, CACTTCATTCTCCCCTTCCATA and GCGATTTAGGTGACACTATAGCATGGAGTCTTGTTTATAGTGGC. *In situs* were performed using the DIG-labeled *Ebf1* probes. The *in situ* product was visualized using NBT/BCIP (Sigma-Aldrich B1911) and anti-DIG conjugated to secondary antibody (Roche, 11093274910). Tissue sections were imaged using a Zeiss Axioplan 2 microscope.

### FACS and single cell RNA-seq

SOX2-GFPhigh^+^ cochlear duct cells were isolated as described previously (Wilkerson et al., 2019) from 10-12 cochleae gathered from one to two litters per stage. Libraries were prepared using the Chromium Single Cell 3′ Library & Gel Bead Kit v3 (10x Genomics) according to the manufacturer's instructions. Reads were aligned to mm10 and filtered using 10X Genomics Cell Ranger, as described previously (Wilkerson et al., 2021). Briefly, E12, E14 and E16 samples were batch-corrected and normalized in Seurat 4 (Butler et al., 2018; Satija et al., 2015). Principal component, UMAP and clustering analysis (McInnes and Healy, 2018 preprint) was performed in Seurat; trajectory analysis was performed in Monocle 3 (Cao et al., 2019). Cell types were identified based on cluster-specific expression of known markers (see Fig. 1). Differential expression analysis was performed on raw counts using the FindMarkers function in Seurat.

### Immunostaining

For embryonic stages, heads were fixed overnight at 4°C. P1 and adult temporal bones were fixed in 4% paraformaldehyde (PFA) either for 30 min at room temperature or overnight at 4°C. After fixation, heads and temporal bones were washed in PBS three times for 10 min on a nutator at room temperature. For wholemount tissue experiments, cochlear ducts were dissected from embryonic heads and P1 temporal bones. For tissue section experiments, the heads and temporal bones were then incubated in a sucrose series consisting of 5%, 10% and 15% sucrose washes. Adult temporal bones and embryonic heads were incubated in an additional 25% sucrose and 30% sucrose wash, respectively. After incubating in OCT (Tissue-Tek 4583) at 4°C, heads and temporal bones were embedded in OCT and cryosectioned at 12 µm.

Wholemount tissue was permeabilized with 0.5% TX-100 for experiments visualizing cell surface markers and 2% Triton X-100 for experiments visualizing nuclear or cytoplasmic markers. Before blocking, adult sections to be stained with rabbit anti-SOX2 antibody were treated with 1% SDS for 5 min. Sections and wholemount tissue were incubated in primary antibodies diluted in block overnight at room temperature. Sections and wholemount tissue were blocked in 10% serum (donkey or fetal bovine) with 0.1% and 0.5% Triton X-100, respectively. Wholemount tissue was washed for 15 min or 1.5 h three times in 0.1% and 0.5% Triton X-100 for sections and wholemount tissue, respectively. Wholemount tissue was subsequently incubated in block for another 1 h. Sections and wholemount tissue were incubated in secondary antibodies diluted in block for 50 min or overnight at room temperature. Antibody details are provided in Table S2. All samples were imaged on a Zeiss LSM 880 confocal microscope.

### FM1-43 uptake

To identify the presence of mechanoelectrical transduction (MET) channels in HCs, we treated freshly dissected P6 cochlear ducts with a styryl dye, FM1-43 FX (4 µM; Thermo Fisher Scientific F35355), for 30 s at room temperature. After removing the FM1-43 dye, the cochlear ducts were washed with HBSS three times. The cochlear wholemounts were imaged on a Zeiss LSM 880 confocal microscope.

### Proliferation assay

To trace proliferation at embryonic stages, we administered 5-ethynyl-2′-deoxyuridine (EdU; 50 mg/kg; Invitrogen A10044) reconstituted in sterile PBS to pregnant dams via intraperitoneal injection twice a day (separated by 8 h) starting on E15 and ending on E17. Click-iT Alexa-Fluor-647 (Invitrogen C10430) was used to detect incorporated EdU in P1 cochlear ducts using the manufacturer's protocol. The EdU-labeled cochlear wholemounts were imaged on a Zeiss LSM 880 confocal microscope.

### Morphometrics

To quantify HCs in *Slc26a9^Ebf1-cKO^* (*Slc26a9^P2A^*^-Cre^ *Ebf1*^fl/fl^) and control (*Ebf1^fl/fl^*) littermates, anti-MYO7a^+^ HCs were counted using the point tool and ROI Manager in ImageJ/Fiji. iHCs were distinguished from oHCs based on their comparatively strong expression of vGLUT3 (SLC17A8) protein and cell shape. Just as the oHCs taper from three rows in the base to two rows in the hook region of *Ebf1^fl/fl^* control cochleae, a reduction in the number of oHC rows can be seen in the hooks of *Slc26a9^Ebf1-cKO^* cochleae. Counts were made starting in the base of each cochlea up to the point in the apex where anti-vGLUT3^+^ iHCs were no longer visible. To measure cochlear duct length, we used the segmented line tool to generate a line composed of 50 µm segments along the space between the iHCs and oHCs. We chose to use this space rather than the row of iPC nuclei (as was done for the *Sox2*-conditional model) because iPCs cannot be identified based on the unique shape of their nuclei in *Slc26a9^Ebf1-cKO^* mice. The segmented line tool was also used to measure the sweep of differentiation in embryonic cochlear wholemounts. We measured from the apex of the cochlear epithelium to the first SOX2^+^ cell in the apex and along medial edge of the strongly SOX2^+^ prosensory domain to the first MYO7a^+^ cell in the apex. For all regional analyses in the cochlea, HC density was measured in ∼400 μm samples in the base, middle and apex. Basal measurements were made starting immediately above the hook. Apical measurements were made in the most apical region of the duct where a space was still evident between the iHCs and oHCs. Middle measurements were made at the approximate midpoint for the duct. For EdU^+^ counting in HCs, EdU^+^ iHCs were distinguished from EdU^+^ oHCs based on vGLUT3 protein expression and cell shape.

To quantify HCs in *Sox2^Ebf1-cKO^* (*Sox2^CreER^ Ebf1^fl/fl^* treated with tamoxifen) mice, littermate controls (*Ebf1^fl/fl^* treated with tamoxifen) and *Sox2^CreER^ Ebf1*^fl/fl^ mice not treated with tamoxifen, anti-MYO7a^+^ HCs were counted using the point tool and ROI Manager in ImageJ/Fiji. The hook region was excluded from counting and length measurements. Counts were made starting in the base of each cochlea, immediately above the hook, up to the point in the apex where iHCs were missing and intermittent (i.e. ∼10% distance from the apex in E18). To quantitate HC density, the length of the counted region was measured using the segmented line tool to measure between iHCs and oHCs, along the zipper-like row of anti-SOX2^+^ PC nuclei. To measure total cochlear duct length, the segmented line tool was used to draw a line along the PC region. For regional analysis of iHC density in the cochlea, HC density was measured in ∼300 μm samples in the base, middle and apex. Basal measurements were made starting immediately above the hook (90-100% distance from the apex). Apical measurements were made from the point in the apex where iHCs were missing and intermittent, extending toward the base (10-20% distance from the apex). Middle measurements were made at ∼40-50% distance from the apex. For regional analysis of oHC density in the cochlea, HC density was measured in ∼100 μm samples in the base, middle and apex. oHC differentiation slightly lags iHC differentiation at E18, so apical oHC counts were made at ∼20-30% distance from the apex in regions containing three or more rows of oHCs. Basal and middle oHC counts were made in approximately the same regions as the iHC counts in each image.

### Auditory brainstem response

Mice were anesthetized by intraperitoneal injection of ketamine (90 mg/kg) and xylazine (10 mg/kg). Subcutaneous electrodes were placed at the cranial vertex for recording, behind the pinna on the stimulus side for reference and behind the contralateral pinna for grounding. Mice were placed in a sound-attenuating chamber and then calibrated tone pips (at 6, 12, 18, 24, 32 45 and 60 kHz; 500 responses at 21/s) and broadband click stimuli (300 responses, at 42.6/s) were presented and the responses recorded from 0-90 dB SPL (5 dB steps) using an RZ6 system (Tucker Davis Technologies). ABR thresholds were determined manually by experienced readers as the minimum sound level, eliciting identifiable ABR waveforms compared with the 0 dB level.

### Experimental design and statistical analyses

The number of animals analyzed per genotype and/or treatment group is listed under sample size in the figure legend for each experiment as well as in Table S1. Biological replicates were analyzed in expression experiments. Male and female mice were used for all experiments.

Graphs were created in GraphPad Prism. Mean total HC counts, HC and EdU^+^ HC densities, and sweep of differentiation are shown as bar graphs with standard deviation error bars. Mean ABR thresholds are shown as a line graph with standard deviation error bars. Significance is indicated as follows: *P<0.05, **P<0.01, ***P<0.001 and ****P<0.0001.

For duct length, total HC counts, PRDM16 and SOX2 expression areas, and sweep of differentiation, statistically significant differences between genotypes were identified using Welch's *t*-tests. Two-way ANOVA with Holm-Šídák's multiple comparisons test was used to test statistically significant differences in HC and EdU^+^ HC density between *Slc26a9^Ebf1-cKO^* and littermate control samples for each region analyzed (base, middle and apex). To test for statistically significant differences between regions in these genotypes, two-way repeated measures ANOVA was performed with the same multiple-comparisons correction. Two-way ANOVA with Holm-Šídák's multiple comparisons test was also used to determine statistically significant differences in ABR thresholds between adult *Slc26a9^Ebf1-cKO^* and control littermates for each stimulus frequency. Two-way ANOVA with Tukey's multiple comparisons test was used to test for statistically significant differences in HC density between samples from *Sox2^Ebf1-cKO^* mice, littermate controls treated with tamoxifen and *Sox2^CreER^ Ebf1*^fl/fl^ mice not treated with tamoxifen. To test for statistically significant differences between regions within these genotypes, two-way repeated measures ANOVA was performed with the same multiple-comparisons correction (Table S1).

## Supplementary Material



10.1242/develop.202816_sup1 Supplementary information
