# Supplementary material for: Deletion of the Ebf1, a mouse deafness gene, causes a dramatic increase in hair cells and support cells of the organ of Corti
Source: Development. 2024 Aug 20;151(16):dev202816. doi: 10.1242/dev.202816 (PMC11361633; doi:10.1242/dev.202816)
Supplement: Supplementary information [file develop-151-202816-s1.pdf]

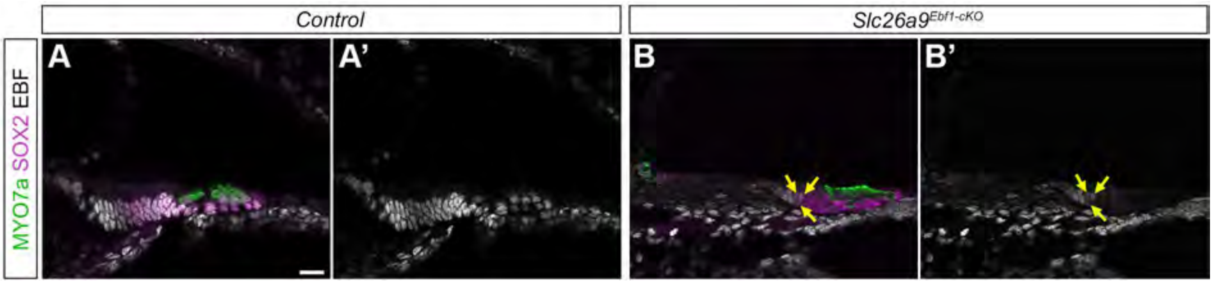

Fig. S1. *Ebf expression is reduced in Slc26a9$^{Ebf1\text{-}cKO}$ cochleae.* ***A-B'*** Sections capturing the base of P1 control and *Slc26a9$^{Ebf1\text{-}cKO}$* cochleae. ***A and A'***, EBF expression is present in HCs, SCs, Kölliker's organ cells, mesenchymal cells, and Schwann cells of control cochleae. ***B and B'***, *Slc26a9$^{Ebf1\text{-}cKO}$* cochleae lack EBF expression in the HCs and SCs of the sensory domain but EBF^{+} cells can occasionally be observed in the Kölliker's organ (arrows).

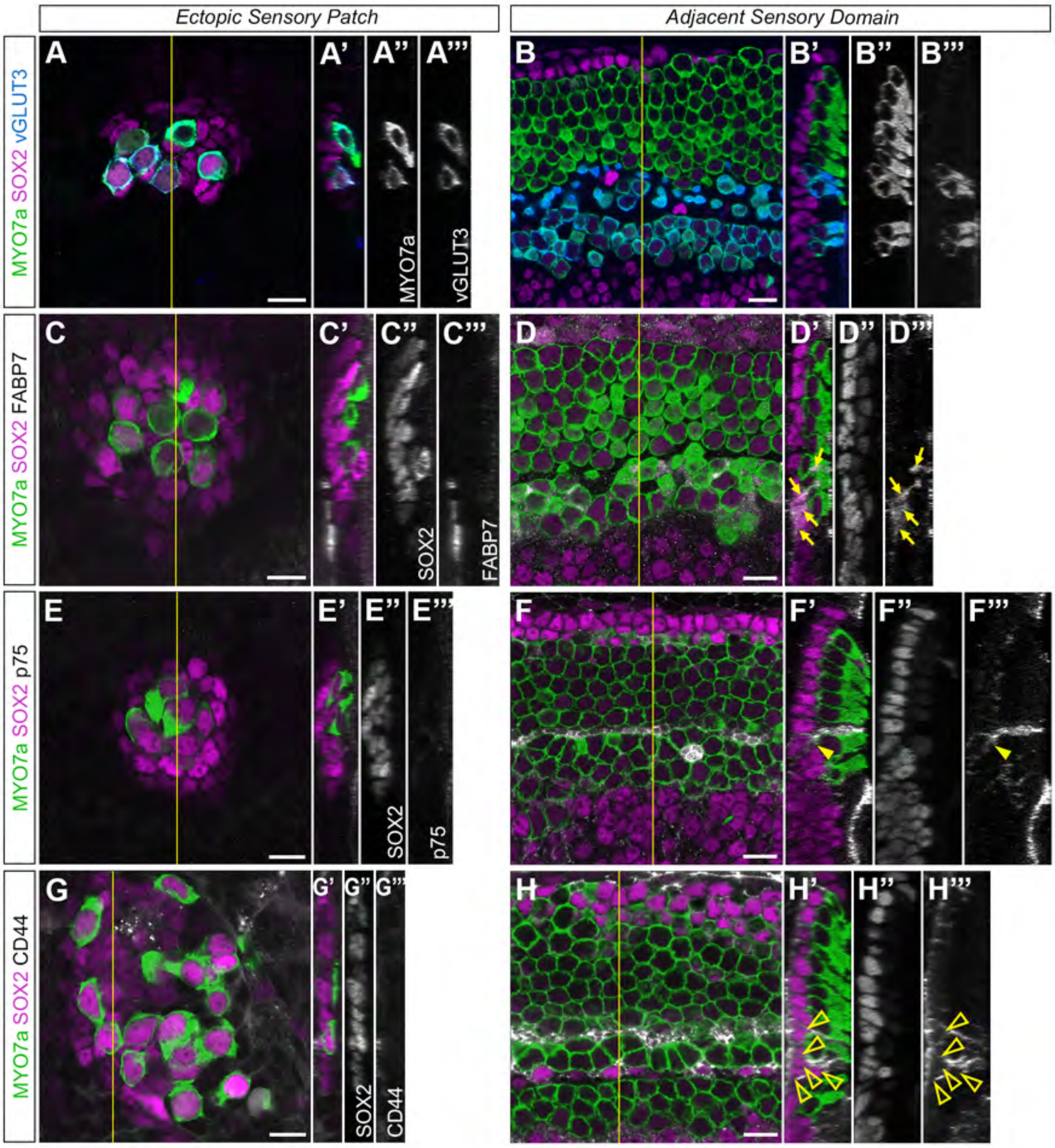

Fig. S2. *HCs in ectopic sensory patches express vGLUT3 while their associated SCs fail to express markers associated with SC subtypes.* ***A-H***, single xy plane confocal images of ectopic sensory patches and the sensory domains adjacent to these patches in P1 *Slc26a9*$^{Ebf1\text{-}cKO}$ cochlear wholemounts. ***A’-H’***, orthogonal projections of the yz plane (yellow line in xy plane images) of ectopic sensory patches and the adjacent sensory domains. ***A-B’’’***, HCs in ectopic sensory patches are vGLUT3^{+}. vGLUT3^{+} iHCs can be seen on either side of the PC region in the *Slc26a9*$^{Ebf1\text{-}cKO}$ sensory domain. ***C-D’’’***, SCs in ectopic sensory patches do not express FABP7. FABP7^{+} iBCs/iPhCs can be seen in the neighboring portion of the *Slc26a9*$^{Ebf1\text{-}cKO}$ sensory domain. ***E-F’’’***, SCs in ectopic sensory patches do not express p75. p75^{+} iPCs can be seen in neighboring portion of the *Slc26a9*$^{Ebf1\text{-}cKO}$ sensory domain. ***G-H’’’***, SCs in ectopic sensory patches do not express CD44. CD44^{+} oPCs can be seen in neighboring portion of the *Slc26a9*$^{Ebf1\text{-}cKO}$ sensory domain. Scale bars: 10 µm for A (same for A’-A’’’), B (same for B’-B’’’), C (same for C’-C’’’), D (same for D’-D’’’), E (same for E’-E’’’), F (same for F’-F’’’), G (same for G’-G’’’), and H (same for H’-H’’’).

Development • Supplementary information

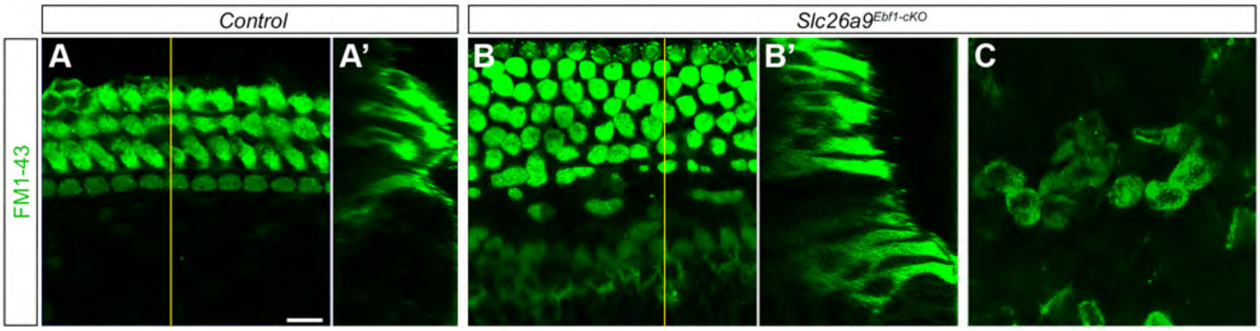

Fig. S3. *Supernumerary HCs in the sensory domain and HCs in the ectopic sensory patches label with FM1-43 dye.* ***A and B***, single xy plane confocal images of middle region of P6 control and *Slc26a9*$^{Ebf1\text{-}cKO}$ cochlear wholemounts. ***A' and B'***, orthogonal projections of the yz plane (yellow line in xy plane images) for the control and *Slc26a9*$^{Ebf1\text{-}cKO}$ cochlear wholemounts. ***A and A'***, FM1-43 labeling in the sensory domain of a control cochlear wholemount. ***B-C***, FM1-43 labeling in the sensory domain and an ectopic sensory patch of a *Slc26a9*$^{Ebf1\text{-}cKO}$ cochlear wholemount. Scale bars: 10 µm for A (same for A' and B-C).

Development • Supplementary information

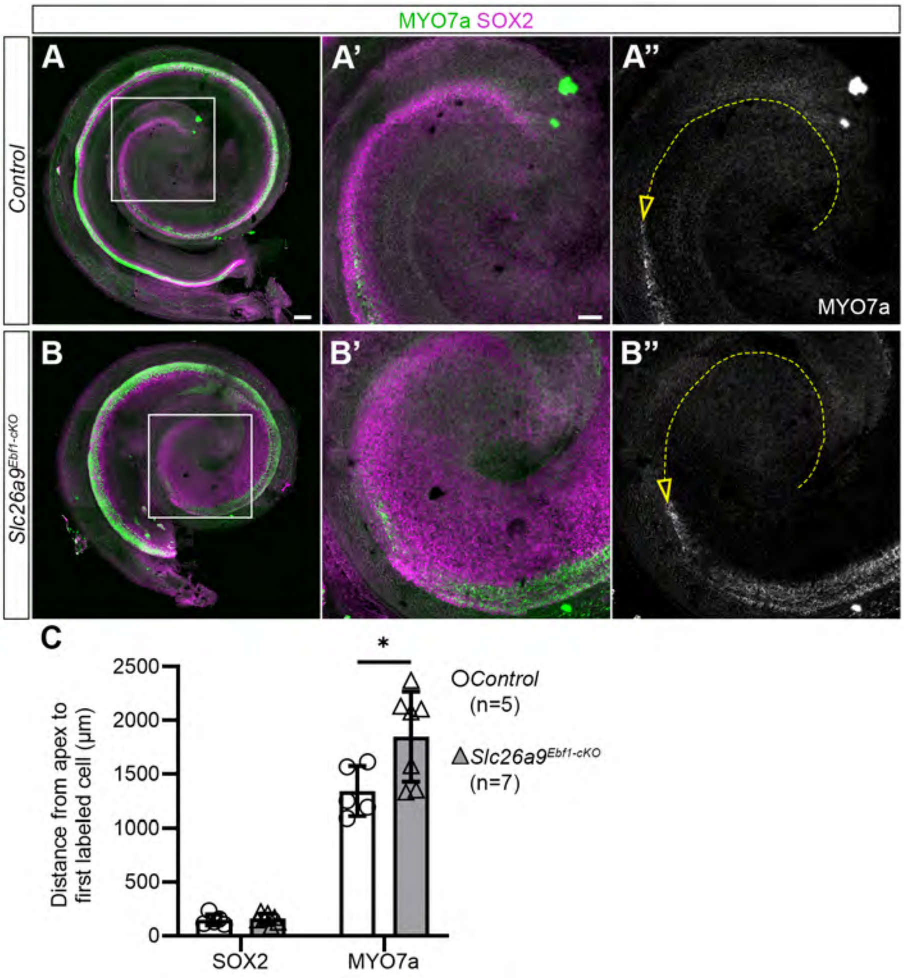

Fig. S4. *Delayed sensory cell differentiation in embryonic Slc26a9$^{Ebf1\text{-}cKO}$ cochleae.* ***A-B,*** E16.5 control and *Slc26a9$^{Ebf1\text{-}cKO}$* cochlear wholemounts. ***A'-B'',*** Zoomed view of apex in the control and *Slc26a9$^{Ebf1\text{-}cKO}$* cochlear wholemounts (boxes in A and B). ***A'' and B'',*** The sweep of differentiation was quantified in cochlear wholemounts by measuring the distance (yellow dashed line in A'' and B'') from the apex of the cochlear epithelium to the first cell that expressed either SOX2 or MYO7a (open arrowhead). ***C***, Compared to cochleae from E16.5 control littermates, the base-to-apex sweep of MYO7a expression is significantly delayed in *Slc26a9$^{Ebf1\text{-}cKO}$* cochleae (Table 2). Sample size: five control and seven *Slc26a9$^{Ebf1\text{-}cKO}$* mice from two litters, one duct per mouse. Scale bars: 100 µm for A (same for B) and 50 µm for A' (same for A'', B', and B'').

Table S1. Statistical analyses

Comparison	***Parameter***	***Sample size***	***Statistical test***	***Significant p-values***
P1 *Slc26a9*$^{Ebf1\text{-}cKO}$ vs. littermate control	Total HC count	6 *Slc26a9*$^{Ebf1\text{-}cKO}$ and 4 control mice from 2 litters, 1 duct per mouse	Welch's t-test 2-tailed	iHCs: p < 0.0001<br>oHCs: p < 0.0001
P1 *Slc26a9*$^{Ebf1\text{-}cKO}$ vs. littermate control	HC density	6 *Slc26a9*$^{Ebf1\text{-}cKO}$ and 4 control mice from 2 litters, 1 duct per mouse	Two-way ANOVA with Holm-Šídák's multiple comparisons test	iHCs: p < 0.0001 for all regional comparisons<br>oHCs: p < 0.0001 for base and middle, p = 0.0008 for apex
P1 *Slc26a9*$^{Ebf1\text{-}cKO}$ vs. littermate control	Area of marker expression	6 *Slc26a9*$^{Ebf1\text{-}cKO}$ and 6 control mice from 4 litters, 1 tissue section per mouse	Welch's t-test 2-tailed	SOX2: p = 0.0382<br>PRDM16: p = 0.0213
P1 *Slc26a9*$^{Ebf1\text{-}cKO}$ vs. littermate control	EdU$^+$ HC density	5 *Slc26a9*$^{Ebf1\text{-}cKO}$ and 6 control mice from 2 litters, 1 duct per mouse	Two-way ANOVA with Holm-Šídák's multiple comparisons test	EdU$^+$ iHCs: p = 0.0009 for base, p = 0.0003 for middle, p < 0.0001 for apex<br>EdU$^+$ oHCs: p = 0.0002 for base, p = 0.0331 for middle, p < 0.0001 for apex
Regions within P1 *Slc26a9*$^{Ebf1\text{-}cKO}$	HC density	6 *Slc26a9*$^{Ebf1\text{-}cKO}$ mice from 2 litters, 1 duct per mouse	Two-way repeated measures ANOVA with Holm-Šídák's multiple comparisons test	iHCs: p = 0.0084 for base vs. middle, p = 0.0084 for base vs. apex<br>oHCs: p = 0.0096 for base vs. middle, p = 0.0013 for base vs. apex, p = 0.0108 for middle vs. apex
Regions within P1 *Slc26a9*$^{Ebf1\text{-}cKO}$	EdU$^+$ HC density	5 *Slc26a9*$^{Ebf1\text{-}cKO}$ and 6 control mice from 2 litters, 1 duct per mouse	Two-way repeated measures ANOVA with Holm-Šídák's multiple comparisons test	EdU$^+$ oHCs: p = 0.0261 for base vs. middle, p = 0.0392 for base vs. apex, p = 0.0261 for middle vs. apex
E16.5 *Slc26a9*$^{Ebf1\text{-}cKO}$ vs. littermate control	Base-to-apex sweep of marker expression	7 *Slc26a9*$^{Ebf1\text{-}cKO}$ and 5 control mice from 2 litters, 1 duct per mouse	Welch's t-test 2-tailed	p = 0.0242
E16.5 *Slc26a9*$^{Ebf1\text{-}cKO}$ vs. littermate control	Duct length	7 *Slc26a9*$^{Ebf1\text{-}cKO}$ and 5 control mice from 2 litters, 1 duct per mouse	Welch's t-test 2-tailed	p = 0.0029
P50-74 *Slc26a9*$^{Ebf1\text{-}cKO}$ vs. littermate control	ABR thresholds	9 *Slc26a9*$^{Ebf1\text{-}cKO}$ (6 females and 3 males) and 5 control (1 female and 4 males) mice from 3 litters	Two-way ANOVA with Holm-Šídák's multiple comparisons test	p < 0.0001 for 6, 12, 18, 24, 30, and 45 kHz, p < 0.0004 for 60 kHz
E18 *Sox2*$^{Ebf1\text{-}cKO}$ vs. *Sox2*^{CreER}-negative littermate control	HC density	14 *Sox2*$^{Ebf1\text{-}cKO}$ and 15 *Sox2*^{CreER}-negative control mice from 6 litters, 1 duct per mouse	Two-way ANOVA with Tukey's multiple comparisons test	iHCs: p < 0.0001 for middle and apex<br>oHCs: p < 0.0001 for apex
Regions within E18 *Sox2*$^{Ebf1\text{-}cKO}$	HC density	14 *Sox2*$^{Ebf1\text{-}cKO}$ from 6 litters, 1 duct per mouse	Two-way repeated measures ANOVA with Tukey's multiple comparisons test	iHCs: p < 0.0001 for base vs. middle and base vs. apex<br>oHCs: p = 0.0001 for base vs. middle, p = 0.0026 for base vs. apex
Regions within E18 *Sox2*^{CreER}-negative controls	HC density	15 *Sox2*^{CreER}-negative control mice from 6 litters, 1 duct per mouse	Two-way repeated measures ANOVA	iHCs: p < 0.0001 fir base vs. middle, p = 0.0001 for base vs. apex<br>oHCs: p < 0.0001 for base vs. middle, p = 0.0049 for base vs. apex

Development • Supplementary information

Table S2. Antibodies used for immunostaining

Antigen	Host	Dilution	Cat. No.	Vendor
CD44	Rat	1:600	14-0441-82	Thermo fisher
EBF	Mouse	1:200	sc167065-af647	Santa Cruz
FABP7	Rabbit	1:1000	ab32423	Abcam
JAGGED1	Rabbit	1:125	2620	Cell signaling
MYO7a	Rabbit	1:1000	25-6790	Proteus
MYO7a	Guinea pig	1:1000	n/a	Stefan Heller
Neurofilament	Chicken	1:200	ab134458	Abcam
PRDM16	Sheep	1:200	af6295	R&D systems
Prestin	Goat	1:200	sc22692	Santa Cruz
PROX1	Rabbit	1:1000	ab5475	Chemicon
SOX2	Goat	1:200	af2018	R&D systems
SOX2	Rabbit	1:200	ab97959	Abcam
vGLUT3	Rabbit	1:300	135203	Synaptic systems
