# Supplementary material for: Deletion of the Ebf1, a mouse deafness gene, causes a dramatic increase in hair cells and support cells of the organ of Corti
Source: Development. 2024 Aug 20;151(16):dev202816. doi: 10.1242/dev.202816 (PMC11361633; doi:10.1242/dev.202816)
Supplement: Supplementary file 1 [file develop-151-202816_review_history.pdf]

Ebf1 is a mouse deafness gene and deletion causes a dramatic increase in hair cells and support cells of the organ of Corti

Kathryn G. Powers, Brent Wilkerson, Kylie E Beach, Sophie S. Seo, Jose Severiano-Rodrigues, Ashton N. Baxter, Sarah E. Hunter and Olivia Bermingham-McDonogh
DOI: 10.1242/dev.202816

Editor: Francois Guillemot

Review timeline
Original submission: 21 February 2024
Editorial decision: 8 April 2024
First revision received: 31 May 2024
Accepted: 5 July 2024

Original submission

First decision letter

MS ID#: DEVELOP/2024/202816

MS TITLE: Ebf1 is a mouse deafness gene and deletion causes a dramatic increase in hair cells and support cells of the organ of Corti

AUTHORS: Kathryn G Powers, Brent Wilkerson, Kylie E Beach, Sophie S Seo, Jose Severiano-Rodrigues, Ashton N Baxter, and Olivia Bermingham-McDonogh

I have now received the reports of three referees on your manuscript and I have reached a decision. The referees' comments are appended below, or you can access them online: please go to BenchPress and click on the 'Manuscripts with Decisions' queue in the Author Area.

As you will see, all the referees are enthusiastic about your work, and we would like to publish a revised manuscript in Development, provided that you satisfactorily address all the suggestions and comments of the referees. Please attend to all these comments in your revised manuscript and detail them in your point-by-point response. If you do not agree with any of their criticisms or suggestions explain clearly why this is so. If it would be helpful, you are welcome to contact us to discuss your revision in greater detail. Please send us a point-by-point response indicating your plans for addressing the referees' comments, and we will look over this and provide further guidance.

Reviewer 1

Advance summary and potential significance to field

The study by Powers et al., investigates the role of the transcription factor EBF1 in the development of the auditory-sensory organ (cochlea). The authors show that Ebf1 is highly expressed in cochlear non-sensory epithelial cells (Kölliker's cells) and pro-sensory cells, and that EBF1 expression declines as pro-sensory cells differentiate into hair cells and supporting cells. Generating otic-specific Ebf1 knockout mice the authors show that loss of EBF1 function at an early otic stage (E9.5) leads to a doubling of the size of the cochlear sensory epithelium and the appearance of ectopic sensory patches within KöllikerÂ's cell region (medial to sensory domain). The authors go on to show that the observed increase in the number of hair cells and supporting

© 2024. Published by The Company of Biologists under the terms of the Creative Commons Attribution License (https://creativecommons.org/licenses/by/4.0/).

cells is in part due to prolonged cell proliferation caused by a delay in pro-sensory cell cycle exit. Despite a mild delay in the onset of cell differentiation marker analysis shows that loss of Ebf1 does not disrupt cell type-specific differentiation and maturation program of hair cells and supporting cells.
However, neuronal innervation is highly abnormal and Ebf1 mutant mice are deaf.
Interestingly, later deletion of Ebf1 at E11.5-E12.5 in pro-sensory cells using Sox2-CreER results only in mild overproduction of inner hair cells.

EBf1 is most often viewed as a pro-differention factor. However, the author's novel findings suggest that in the developing cochlea EBF1 function is dispensable for cell differentiation but is required for pro-sensory domain formation/patterning and limiting cell proliferation.

Comments for the author

On the whole, this is a well-designed and executed study, the main conclusions are all well supported. I have only few minor comments/ critiques:

1) The authors show that the number of inner pillar cells does no double as shown for other supporting cell subtypes (e.g. outer pillar cells). This is quite interesting, however it relies on a single marker for inner pillar cells (p75). There are several other markers such as Npy (and Lgr6) the authors could use to determine whether inner pillar cell development is unaffected by the loss of Ebf1.

2) The authors state that the number of PRDM16+ Kölliker's cells increased in Ebf1 cKO mice. The authors should quantify this.

3) Does Ebf1 have a function in the development of spiral ganglion neurons (SGNs)? The authors state that EBF1 is expressed in a subset of neurons and the authors show that Ebf1 cKO mice have severe defects in hair cell innervation.
The used strategy does knockout Ebf1 in developing SGNs. The authors should discuss possible ramifications.

4) In Figure6 the images of the immuno-stained cochlear tissue are taken at low power making it hard to identify the various cell types within the organ of Corti. Additional high power images would be helpful.

5) The authors show that conditional deletion of Ebf1 in pro-sensory cells at E11.5-E12.5 (Sox2-CreER) results only in a mild increase in the number of inner hair cells. The authors should include data that shows that Ebf1 was successfully deleted using Sox2-CreER strategy.

Additional minor comments:

Figure legend figure 1, A and B label are switched.

Reviewer 2

Advance summary and potential significance to field

How the cochlea is patterned into precise rows of hair cells and supporting cells is not well understood. This manuscript describes the role of a new transcription factor in cochlear development, Ebf1. The authors describe its expression pattern during cochlear development using a variety of methods including single cell RNAseq, in situ hybridization and immunohistochemistry. These studies reveal an expression pattern of strong medial expression in the floor of the cochlea (Kolliker's organ), as well as mesenchymal and spiral ganglion expression.

Deletion using an epithelial specific Cre, Slc26a, results in an expanded sensory region, with multiple rows of both inner and outer hair cells, as well as supporting cells. The authors show that the increased sensory cell region is likely due to delayed differentiation and continued proliferation. The authors also show that the supernumerary hair cells and supporting cells are still

© 2024. Published by The Company of Biologists under the terms of the Creative Commons Attribution License (https://creativecommons.org/licenses/by/4.0/).

present in the adult Ebf1 mutant cochlea and the mice are deaf. The experiments are well performed and quantified. Overall these data introduce a new player in the regulation of the cell cycle and sensory differentiation in the mammalian cochlea.

Comments for the author

Specific comments

The ectopic sensory regions are quite medial in the wholemount cochlea (Fig. 1C-D), appearing almost beyond the Kolliker's region. Is it possible these ectopic regions are in the ganglia? Showing these regions in section would help clarify their position.

The authors suggest that in the absence of Ebf1, the sensory region is slow to exit the cell cycle, and that may explain the extra sensory cells. While the paper shows that there are cells that continue to proliferate after normal cell cycle exit, they do not show whether there is a delay in cell cycle exit at the earliest time point (E12-E14). Moreover, the delayed differentiation shown in Fig S4 is somewhat subtle. The delayed exit could be shown by determining if P27KIP1 (Cdkn1b) expression shows delayed onset apex to base, and/or whether the zone of non-proliferation is delayed. Given the more moderate phenotype when Ebf1 is deleted at E11, these results indicate that Ebf1 is playing a significant role prior to E12.

It's unclear exactly where the extra HC and SC are coming from. Are they coming from Kolliker's organ? Are they coming from organ of Corti cells? The authors suggest that the role of Ebf1 is regulating exit from the cycle. However, since the cells in Kolliker's organ are already cycling throughout cochlear development it's unclear what the role is for Ebf1 would be in this structure.

The effects on the spiral ganglion cells are not clear—is Ebf1 deleted in this structure? Is the ganglion a normal size? There appears to be increased fibers in Fig. 7. It is unclear if the defects in innervation are cell autonomous or not.

The results of the ABR analysis are not entirely clear, are there no responses at the highest sound levels? If so, this should be indicated in some way in the text and on the figure.

The images depicting the adult organ of Corti are difficult to see (fig. 6), given the low power images. While it is nice to see the other structures, the organ of Corti is very small and it is difficult to see the differences in the mutant cochlea. Particularly, it is hard to see the multiple tunnels of Corti in the mutant.

<u>Reviewer 3</u>

Advance summary and potential significance to field

The auditory epithelium is a powerful model system for cell fate specification, with a well-characterised interplay of multiple signalling molecules, transcription factors, specifying cell fate in different domains of the organ of corti. This study investigates the role of the transcription factor Ebf1 in patterning within the structure. The authors find that Ebf1 is expressed in part of the cochlear duct, the spiral ganglion and associated mesencyme. Mutant analysis reveals an explanded sensory domain, with ectopic hair cells that are innervated although the mice are deaf. The study is carefully performed and although a similar study from Norio Yamamoto's lab in Kyoto have also characterised Ebf1, this study present new aspects of the phenotype that may be of significance.

Comments for the author

Figure 1 details the expression domain of Ebf1. Firstly, panel A is mislabelled (in the legend). It would be The expression domain of Ebf1 extends into the peri-otic mesenchyme. A comment about its possible function would be useful. The figure also shows the overlap between Sox2 and Ebf1. Ebf1 extends further than the Sox2 domain - a comment about this would be useful. What are the possible controls for the medial expression? Kagoshima et al., seem to suggest that Ebf1 and Sox2

© 2024. Published by The Company of Biologists under the terms of the Creative Commons Attribution License (https://creativecommons.org/licenses/by/4.0/).

do overlap in the cristae. This observation can also be incorporated in the discussion and commented upon.

Figure 2 is an overview of the phenotype of the conditional knockout. This is one area in which the current manuscript and that from Kagoshima can be distinct: using a slightly earlier Cre gives a slightly different phenotype. It would be worth comparing these phenotypes more explicitly. The authors do quantitate teh extent of knockout, and see traces of Ebf1 in Kollikers Organ - this is possibly confunding, and a comment on whether this is seen in other Cre lines (Pax2-Cre for example) would be good. One of the most interesting phenotype is the discordance between IHC and OHC along the base to apex axis. In particular, having OHC and IHC mixing is uncommon. It would be good to comment on perturbations in bundle polarity as well. The authors can use PCP Autocount (in BioRxiv) to do this quantification. When taken with the p75 expression (Fig 4), this seems to suggest that these two territories are perturbed. Are there any other mutants in which the IHC and OHC domains mix like this?

Figure 3+4: Describes the phenotype of the support cells, and the ability of supernumary iHC to pattern SC associated with IHC. The converse is also interesting - do OHC that find themselves in the IHC domain associate with Deiter's cells. Prox1 is a Deiter cells marker, it would be useful to see if Prox1 is now found ectopically.

Figure 5: This figure shows continued proliferation in the sensory region in the mutants. This is slightly confusing. The authors show (in fig. 8) that a later KO of Ebf1 results in a milder phenotype. Do these cochlea also retain proliferation in the sensory regions?

Figure 6: Characterises the deafness phenotype. As the strain (C57/BL6) is prone to age-related hearing loss the mutant may just exacerbate this decline. Have the authors looked at ABR in younger (P21?) mice? Do the authors observe circling behaviours? Are the Sox2-CreEr mice deaf?

Figure 7: Looks at the innervation phenotype. Innervation in mutants seems to be abundant and indiscriminate. Is there an identification of what is happening to glia in these mutants, as these ar emeant to provide some kind of guide for neuronal migration.

Fig 8: This describes the later effect of deletion. It does raise some quesitons, particularly about the proliferation (as described above) and deafness. In addition, it would be useful to look at the neuronal phenotype here, to ask if these can be are temporally decoupled.

First revision

Author response to reviewers' comments

Response to Reviewers:

Reviewer’s comments in black text and author’s response in blue text.

Reviewer 1 Comments for the Author:

On the whole, this is a well-designed and executed study, the main conclusions are all well supported. I have only few minor comments/ critiques:

1) The authors show that the number of inner pillar cells does no double as shown for other supporting cell subtypes (e.g. outer pillar cells). This is quite interesting, however it relies on a single marker for inner pillar cells (p75). There are several other markers such as Npy (and Lgr6) the authors could use to determine whether inner pillar cell development is unaffected by the loss of Ebf1.

p75 is a highly specific marker for inner pillar cells with CD44 specific for outer pillars at the

© 2024. Published by The Company of Biologists under the terms of the Creative Commons Attribution License (https://creativecommons.org/licenses/by/4.0/).

neonatal ages studied so we chose these two markers to distinguish the pillars. In our wholemount immunolabeling experiments (Fig. 4), we do not see evidence of medial Sox2+ SCs that lack FABP7, p75, or CD44 expression, and consequently, we do not have reason to think that we are missing iPCs with our anti-p75 labeling.

2) The authors state that the number of PRDM16+ Kölliker's cells increased in Ebf1 cKO mice. The authors should quantify this.
What we see is an overall increase in size of both the prosensory domain and the Köllikers' organ. We have added area measurements for the SOX2 and PRDM16 expression domains to the text in the results section.

3) Does Ebf1 have a function in the development of spiral ganglion neurons (SGNs)? The authors state that EBF1 is expressed in a subset of neurons and the authors show that Ebf1 cKO mice have severe defects in hair cell innervation. The used strategy does knockout Ebf1 in developing SGNs. The authors should discuss possible ramifications.

We do not see any obvious loss or increase in spiral ganglion cells. However, the reviewer is correct in pointing out that we do not know that there is not a defect in innervation induced by loss of Ebf1 in the ganglion. To assess this function would require a specific deletion of Ebf1 in ganglion cells.

4) In Figure6 the images of the immuno-stained cochlear tissue are taken at low power making it hard to identify the various cell types within the organ of Corti. Additional high power images would be helpful.

We now provide higher mag images of the organ of Corti for the adult sections.

5) The authors show that conditional deletion of Ebf1 in pro-sensory cells at E11.5- E12.5 (Sox2-CreER) results only in a mild increase in the number of inner hair cells.The authors should include data that shows that Ebf1 was successfully deleted using Sox2- CreER strategy.

Sox2-CreER has been used to delete many genes during cochlear development and has been shown to successfully lineage trace from a Rosa locus after a single injection of tamoxifen. There is also a description of Sox2 and Cre localized to the same cells (Gu et al, Dev. Biol 2016).

Additional minor comments:
Figure legend figure 1, A and B label are switched.

We have corrected our figure legend.

Reviewer 2 Comments for the Author:
Specific comments

The ectopic sensory regions are quite medial in the wholemount cochlea (Fig. 1C-D), appearing almost beyond the Kolliker's region. Is it possible these ectopic regions are in the ganglia? Showing these regions in section would help clarify their position.

The randomness of position and number of ectopic patches make it difficult to capture in sections. However, we have seen some in sections and indeed they can be extremely medially but are never seen in the ganglion itself only in the epithelium.

The authors suggest that in the absence of Ebf1, the sensory region is slow to exit the cell cycle, and that may explain the extra sensory cells. While the paper shows that there are cells that continue to proliferate after normal cell cycle exit, they do not show whether there is a delay in cell cycle exit at the earliest time point (E12-E14). Moreover, the delayed differentiation shown in Fig S4 is somewhat subtle. The delayed exit could be shown by determining if P27KIP1 (Cdkn1b) expression shows delayed onset apex to base, and/or whether the zone of non-proliferation is delayed. Given the more moderate phenotype when Ebf1 is deleted at E11, these results indicate that Ebf1 is playing a significant role prior to E12.

© 2024. Published by The Company of Biologists under the terms of the Creative Commons Attribution License (https://creativecommons.org/licenses/by/4.0/).

We agree with the reviewer that Ebf1 is very likely playing a role very early in development prior to E12. We do see a delay in p27Kip1 onset as one would expect from a delay in cell cycle exit but as we have noted in the text we also observe an overall delay in convergent extension at embryonic ages. Thus, it is difficult to do a direct comparison between cKO and littermate controls as the former might have 2 turns evident while the latter has three.

It's unclear exactly where the extra HC and SC are coming from. Are they coming from Kolliker's organ? Are they coming from organ of Corti cells? The authors suggest that the role of Ebf1 is regulating exit from the cycle. However, since the cells in Kolliker's organ are already cycling throughout cochlear development, it's unclear what the role is for Ebf1 would be in this structure.

The cells are coming from an expansion of the prosensory domain and in addition some co-opting of Köllikers' organ cells. Ebf1 is involved in 2 steps, first in regulating the dropout from the cell cycle and a second, later step in regulating the exact border of the sensory domain medially.

The effects on the spiral ganglion cells are not clear—is Ebf1 deleted in this structure? Is the ganglion a normal size? There appears to be increased fibers in Fig. 7. It is unclear if the defects in innervation are cell autonomous or not.

The ganglion appears normal in size but there may have been some cells that have eliminated Ebf1, however when we look at the ganglion with the pan Ebf antibody at embryonic stages we do not see much Ebf by immunolabeling in the ganglion. We know it is expressed by in situ but the levels of protein are much lower than we see in either the epithelium or the mesenchyme.

The results of the ABR analysis are not entirely clear, are there no responses at the highest sound levels? If so, this should be indicated in some way in the text and on the figure.

There are responses but these can only be detected when the scale on the instrument is adjusted thus at the same scale as one sees response from the control animals the responses from the mutants would be undetectable.

The images depicting the adult organ of Corti are difficult to see (fig. 6), given the low power images. While it is nice to see the other structures, the organ of Corti is very small and it is difficult to see the differences in the mutant cochlea. Particularly, it is hard to see the multiple tunnels of Corti in the mutant.

We now just show the organ of Corti region at higher magnification.

Response to Reviewer's

Reviewer 3 Comments for the Author:

Figure 1 details the expression domain of Ebf1. Firstly, panel A is mislabeled (in the legend). It would be The expression domain of Ebf1 extends into the peri-otic mesenchyme. A comment about its possible function would be useful. The figure also shows the overlap between Sox2 and Ebf1. Ebf1 extends further than the Sox2 domain - a comment about this would be useful. What are the possible controls for the medial expression? Kagoshima et al., seem to suggest that Ebf1 and Sox2 do overlap in the cristae. This observation can also be incorporated in the discussion and commented upon. Figure 2 is an overview of the phenotype of the conditional knockout. This is one area in which the current manuscript and that from Kagoshima can be distinct: using a slightly earlier Cre gives a slightly different phenotype. It would be worth comparing these phenotypes more explicitly. The authors do quantitate teh extent of knockout, and see traces of Ebf1 in Kollikers Organ - this is possibly confunding, and a comment on whether this is seen in other Cre lines (Pax2-Cre for example) would be good. One of the most interesting phenotype is the discordance between IHC and OHC along the base to apex axis. In particular, having OHC and IHC mixing is uncommon. It would be good to comment on perturbations in bundle polarity as well. The authors can use PCP Autocount (in BioRxiv) to do this quantification. When taken with the p75 expression (Fig 4), this seems to suggest that these two territories are perturbed. Are there any other mutants in which the IHC and OHC domains mix like this?

© 2024. Published by The Company of Biologists under the terms of the Creative Commons Attribution License (https://creativecommons.org/licenses/by/4.0/).

We do see Ebf1 expression in the cristae by immunostaining and in situ but have never detected any vestibular disturbance in the mutant animals. We do not know what is regulating the expression of Ebf1 in the organ of Corti or in Köllikers' organ. In Köllikers' organ it overlaps with Prdm16 but Prdm16 is not expressed in the cells of the developing organ of Corti.

Figure 3+4: Describes the phenotype of the support cells, and the ability of supernumary iHC to pattern SC associated with IHC. The converse is also interesting - do OHC that find themselves in the IHC domain associate with Deiter's cells. Prox1 is a Deiter cells marker, it would be useful to see if Prox1 is now found
ectopically.

We do not find OHCs in the IHC domain only the reverse. When an inner HC is in the OHC domain then it does have IHC specific SCs associated.

Figure 5: This figure shows continued proliferation in the sensory region in the mutants. This is slightly confusing. The authors show (in fig. 8) that a later KO of Ebf1 results in a milder phenotype. Do these cochlea also retain proliferation in the sensory regions?

We have seen some of the extra HCs in the Sox2-Cre mutant labeling with EdU after normal cell cycle exit. This may indicate that after E12 we are only interfering with the position of the medial border of the sensory domain and see a co-option of Kolikers' organ cells into IHC and ISCs. Since this region would normally still be cycling the presence of EdU in the additional IHCs is expected.

Figure 6: Characterises the deafness phenotype. As the strain (C57/BL6) is prone to age-related hearing loss, the mutant may just exacerbate this decline. Have the authors looked at ABR in younger (P21?) mice? Do the authors observe circling behaviours?
Are the Sox2-CreEr mice deaf?

This may be true, but we compare directly with littermates to avoid the confound of loss of HCs. We also analyze at 8 weeks prior to the usual onset of HC loss in this strain background. We have not looked in younger mice. The Sox2-CreER mice are not born so we cannot do the ABR.

Figure 7: Looks at the innervation phenotype. Innervation in mutants seems to be abundant and indiscriminate. Is there an identification of what is happening to glia in these mutants, as these are meant to provide some kind of guide for neuronal migration.

Any markers we have used that also label glia in the ganglion (Fabp7, Sox2) appear to show the glia are present and no readily apparent decrease in numbers.

Fig 8: This describes the later effect of deletion. It does raise some questions, particularly about the proliferation (as described above) and deafness. In addition, it would be useful to look at the neuronal phenotype here, to ask if these can be are temporally decoupled.

Due to the fact that we had to limit our analysis of the Sox2-Cre mutant to E18.5 we did not look at innervation. The outer spiral bundles are forming in late embryonic and the first postnatal week so even in the wt these are only obvious in the base of the cochlea at E18.5. More recently we have varied the time of tamoxifen induction and looked at spiral bundles and realized that even subtle developmental time differences between embryos would affect the extent of outer spiral process formation. In the Slc-Cre mutant with a doubling of the OHCs the disruption of innervation is very obvious.

<u>Second decision letter</u>

MS ID#: DEVELOP/2024/202816

MS TITLE: Ebf1 is a mouse deafness gene and deletion causes a dramatic increase in hair cells and support cells of the organ of Corti

© 2024. Published by The Company of Biologists under the terms of the Creative Commons Attribution License (https://creativecommons.org/licenses/by/4.0/).

AUTHORS: Kathryn G Powers, Brent Wilkerson, Kylie E Beach, Sophie S Seo, Jose Severiano-Rodrigues, Ashton N Baxter, Sarah E Hunter, and Olivia Bermingham-McDonogh
ARTICLE TYPE: Research Article

I am delighted to tell you that your manuscript has been accepted for publication in Development, pending our standard publication integrity checks.

Reviewer 1

Advance summary and potential significance to field

The study by Powers et al., investigates the role of the transcription factor EBF1 in the development of the auditory-sensory organ (cochlea). The authors show that Ebf1 is highly expressed in cochlear non-sensory epithelial cells (Kölliker's cells) and pro-sensory cells, and that EBF1 expression declines as pro-sensory cells differentiate into hair cells and supporting cells. Generating otic-specific Ebf1 knockout mice the authors show that loss of EBF1 function at an early otic stage (E9.5) leads to a doubling of the size of the cochlear sensory epithelium and the appearance of ectopic sensory patches within Kölliker's cell region (medial to sensory domain). The authors go on to show that the observed increase in the number of hair cells and supporting cells is in part due to prolonged cell proliferation caused by a delay in pro-sensory cell cycle exit. Despite a mild delay in the onset of cell differentiation marker analysis shows that loss of Ebf1 does not disrupt cell type-specific differentiation and maturation program of hair cells and supporting cells. However, neuronal innervation is highly abnormal and Ebf1 mutant mice are deaf.
Interestingly, later deletion of Ebf1 at E11.5-E12.5 in pro-sensory cells using Sox2-CreER results only in mild overproduction of inner hair cells.

EBf1 is most often viewed as a pro-differention factor. However, the author's novel findings suggest that in the developing cochlea EBF1 function is dispensable for cell differentiation but is required for pro-sensory domain formation/patterning and limiting cell proliferation.

Comments for the author

I have no further comments. The authors addressed my previous concerns satisfactory.

Reviewer 3

Advance summary and potential significance to field

Comments for the author

Some minor omissions are there, for example the PRDM16 antibody details were not included in the methods.

© 2024. Published by The Company of Biologists under the terms of the Creative Commons Attribution License (https://creativecommons.org/licenses/by/4.0/).
